# Early immune innate hallmarks and microbiome changes across the gut during *Escherichia coli* O157: H7 infection in cattle

**DOI:** 10.1038/s41598-020-78752-x

**Published:** 2020-12-09

**Authors:** Mariano Larzábal, Wanderson Marques Da Silva, Anmol Multani, Lucas E. Vagnoni, Dadin P. Moore, Maia S. Marin, Nahuel A. Riviere, Fernando O. Delgado, Daniel A. Vilte, Matias Romero Victorica, Tao Ma, Luo Le Guan, Paola Talia, Angel Cataldi, Eduardo R. Cobo

**Affiliations:** 1 Agrobiotechnology and Molecular Biology Institute (IABIMO)-CICVyA, National Agricultural Technology Institute (INTA), National Scientific and Technical Research Council (CONICET), Hurlingham, Argentina; 2 Faculty Veterinary Medicine, Production Animal Health, University of Calgary, HSC 2519, 3330 Hospital Dr. NW, Calgary, AB T2N 4N1 Canada; 3 National Scientific and Technical Research Council (CONICET), National Agricultural Technology Institute (INTA), EEA-Balcarce, Balcarce, Argentina; 4 Veterinary Pathobiology Institute (IPVet) CICVyA, National Scientific and Technical Research Council (CONICET), National Agricultural Technology Institute (INTA), Balcarce, Argentina; 5 Department of Agricultural, Food and Nutritional Science, University of Alberta, Edmonton, Canada; 6 Feed Research Institute/Key Laboratory of Feed Biotechnology of the Ministry of Agriculture and Rural Affairs, Chinese Academy of Agricultural Sciences, Beijing, China

**Keywords:** Immunology, Bacterial infection, Pathogens, Intestinal diseases, Diarrhoea, Gastrointestinal models

## Abstract

The zoonotic enterohemorrhagic *Escherichia coli* (EHEC) O157: H7 bacterium causes diarrhea, hemorrhagic colitis, and hemolytic uremic syndrome (HUS) in humans. Cattle are primary reservoirs and EHEC O157: H7; the bacteria predominately inhabit the colon and recto-anal junctions (RAJ). The early innate immune reactions in the infected gut are critical in the pathogenesis of EHEC O157: H7. In this study, calves orally inoculated with EHEC O157: H7 showed infiltration of neutrophils in the lamina propria of ileum and RAJ at 7 and 14 days post-infection. Infected calves had altered mucin layer and mast cell populations across small and large intestines. There were differential transcription expressions of key bovine β defensins, tracheal antimicrobial peptide (*TAP*) in the ileum, and lingual antimicrobial peptide (*LAP*) in RAJ. The main Gram-negative bacterial/LPS signaling Toll-Like receptor 4 (*TLR4*) was downregulated in RAJ. Intestinal infection with EHEC O157: H7 impacted the gut bacterial communities and influenced the relative abundance of *Negativibacillus* and *Erysipelotrichaceae* in mucosa-associated bacteria in the rectum. Thus, innate immunity in the gut of calves showed unique characteristics during infection with EHEC O157: H7, which occurred in the absence of major clinical manifestations but denoted an active immunological niche.

## Introduction

Enterohemorrhagic *Escherichia coli* (EHEC), a subset of Shiga toxin-producing *E coli* (STEC), are zoonotic foodborne pathogens responsible for uncomplicated diarrheal syndromes^1,2^ to severe manifestations, including hemorrhagic colitis (HC), hemolytic uremic syndrome (HUS) and occasionally, death mostly in children^2–4^. EHEC O157: H7 is globally important because it is the most commonly isolated serotype from various outbreaks worldwide^3^. Ingested through contaminated food, EHEC inhabits the gastrointestinal tracts of humans and other homeothermic animals^3^. In cattle, it colonizes the colon and persists in the rectum^5^. Remarkably, this bacterium does not cause systemic diseases in animals^6,7^ as it does in humans^2,4^. However, from a zoonotic perspective, cattle are the principal reservoir of this bacterium^8^ and the ingestion of raw meat is the riskiest route of infection.

Early in the pathogenesis, EHEC O157: H7 interacts with the apical surface of intestinal epithelial cells and releases virulence factors, including Shiga toxins (Stx), LPS, H7 flagellin, long polar fimbriae (Lpf1/Lpf2), hemorrhagic coli pili, and effector proteins that are injected into host cells through a type 3 secretion system (T3SS)^9–11^. The intestinal innate immune response to EHEC O157: H7 is critical during gut colonization and pathogen control. One aspect is the evolutionarily conserved host defense peptides cathelicidins and β-defensins, with a broad spectrum of antimicrobial and immunomodulatory properties^12−14^. While a single cathelicidin is expressed in humans (cathelicidin antimicrobial peptide; CAMP) and mice (cathelicidin-related-antimicrobial-peptide; CRAMP), cattle express multiple cathelicidin genes (at least seven). Cathelicidins are mostly found in granules of granulocytes and epithelial cells^12,13,15,16^. Bovine cathelicidins include cysteine-rich bactenecin (Bac) 1^17^, proline-rich peptides Bac5 and Bac7^18^, tryptophan-rich indolicidin^19^, and α-helical bovine myeloid antimicrobial peptides (BMAP)-27, BMAP-28^20^, and BMAP-34^21,22^. Cattle also produce several types of β-defensins, including tracheal antimicrobial peptide (TAP)^23^ and lingual antimicrobial peptide (LAP), which are abundant in the gut^24,25^. Reported functions of cathelicidins and β-defensins include bacterial killing and immunomodulatory functions such as chemotaxis of leukocytes, epithelial wound repair, and activation of chemokine secretion^12,14^, infer their potential role in the innate immune defense against EHEC O157: H7. However, the extent to which these peptides occur in the gut and their relevance in EHEC colonization in cattle is unknown.

Other incompletely explored innate defenses during EHEC O157: H7 infection are the colonic mucus layer composed of gel-forming glycoprotein mucins MUC2 and MUC5AC, which are secreted by goblet cells^26–28^. Adherence of EHEC to colonic epithelial cells largely depends on the *O*-glycosylation status of the mucus (eg., α-GalNAc)^29^, although early intestinal mucin responses and glycosylation patterns during EHEC O157: H7 infection are unknown. Additionally, mast cells are strategically abundant in the lamina propria of the mucosa and submucosa of the intestines^30^. These cells can rapidly sense pathogenic microbes and release preformed inflammatory mediators^31^. Whereas mast cell populations in the rectums of EHEC O157: H7 colonized calves were not different after 2 weeks of infection^32^, how mast cells respond earlier during the infection onset is undetermined. These innate gut defenses must coexist with the gut microbiota, which has implications on the exclusion of enteric pathogens^33^ and livestock productivity^34^. Thus, to gain insights into the interactions between the gut innate immunity, microbiome, and EHEC O157: H7, this study aimed to explore early innate immune responses and bacterial communities in the intestinal tract of infected calves.

## Results

### EHEC O157: H7 colonized early the intestine of calves

**Figure 1:**
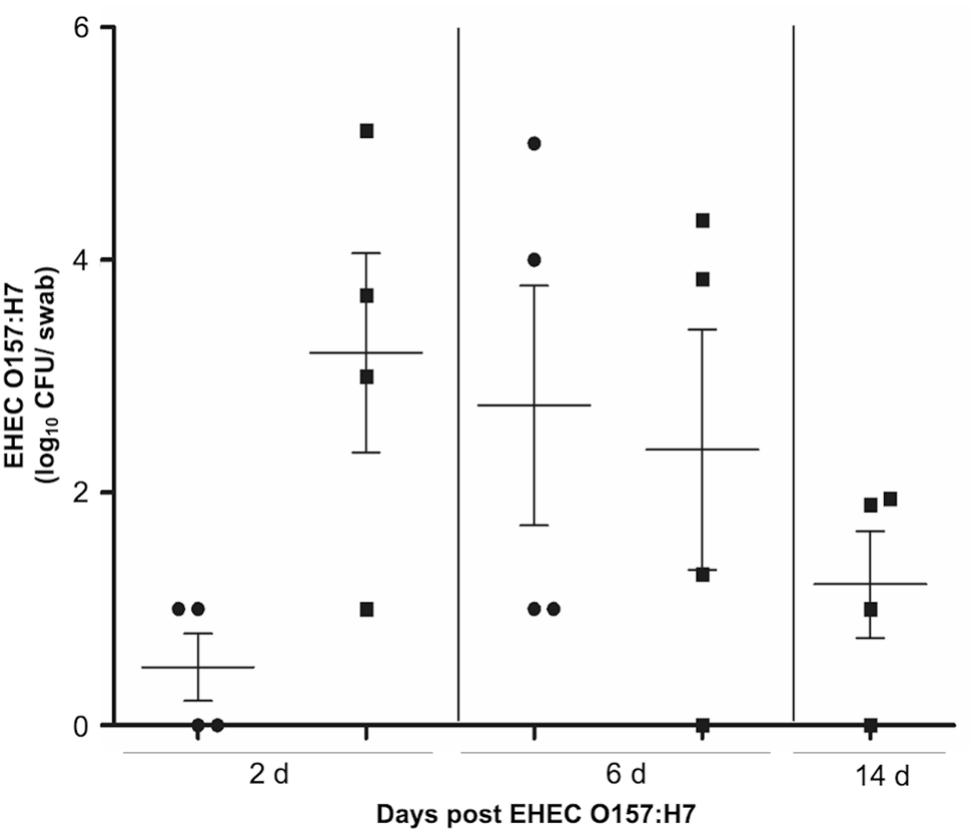
Fecal shedding of EHEC O157: H7 after experimental infection of calves. Circles indicate calves terminated at 7 d post-challenge (Group 1) and squares indicate calves terminated at 14 d post-challenge (Group 2).

To determine gut colonization of EHEC O157: H7 in cattle, calves (n = 8) were inoculated into the rumen with a Shiga toxin-producing strain of EHEC O157: H7 (438/99; *Stx2*^+^, *eae*^+^, and *pO157*^+^; 10^10^ colony-forming unit, CFU) and terminated at 7 d (n = 4; group 1) and 14 d (n = 4; group 2) post-challenge. Of all challenged calves (n = 8), six (6 of 8) shed EHEC O157: H7 at 2 d, and seven (7 of 8) shed EHEC O157: H7 at 6 d post-challenge, as determined by rectal swabs positive to culture and confirmed by multiplex (*stx1, stx2, eae,* and *rbf*_*O157*_) PCR^35^. Most of the calves from group 2 (3 of 4) were still shedding bacteria by 14 d post-challenge (Fig. 1). Uninfected calves did not shed EHEC O157: H7 during the 14-d experimental period (Fig. 1).

### Ileitis with early increased *Il-8* gene expression in EHEC O157: H7 infected calves

**Figure 2:**
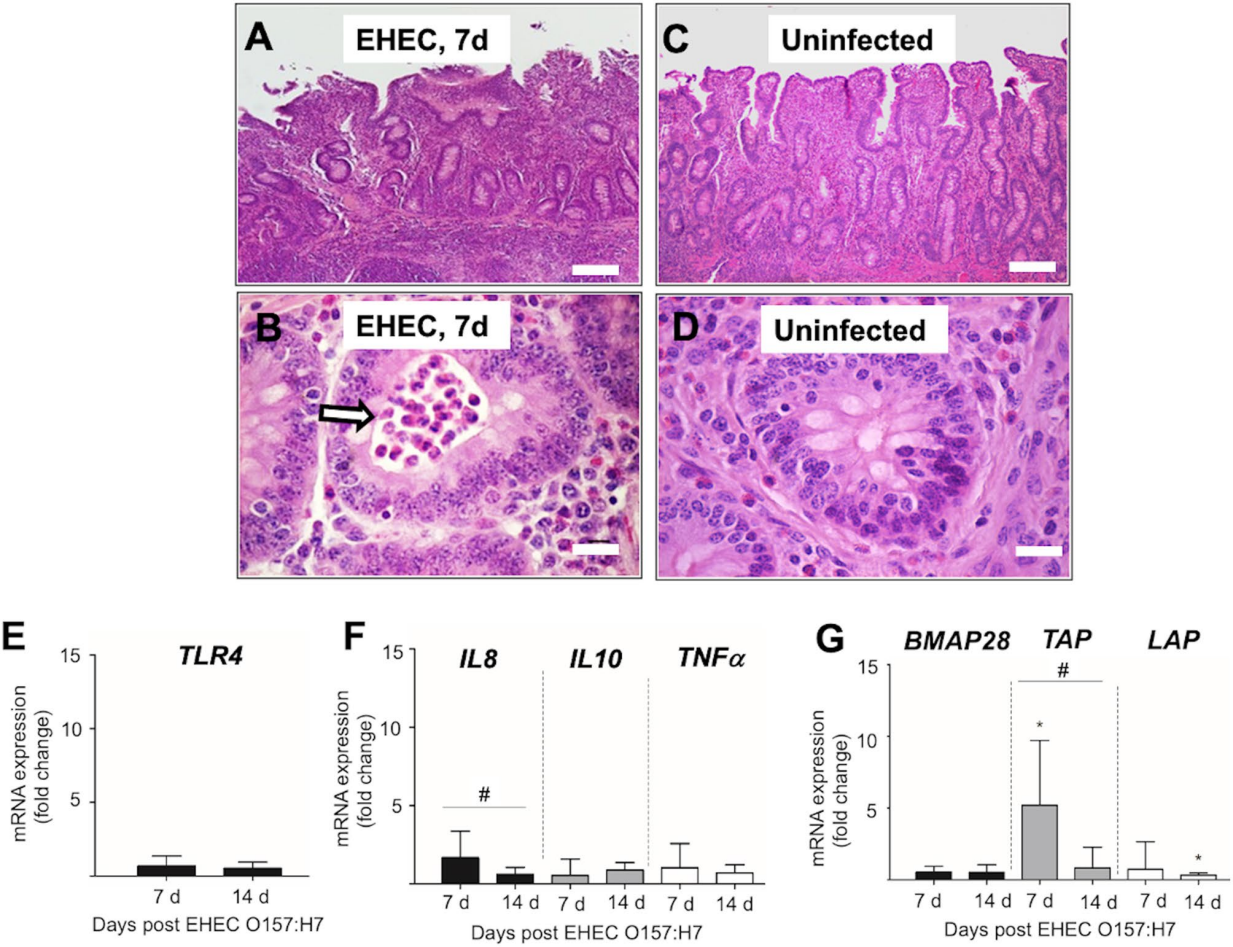
(**A-D**) Microphotographs (hematoxylin and eosin) of ileum from a calf infected with EHEC O157: H7 at 7 d post-challenge showing (**A**) inflammatory infiltrates and disrupted epithelium and atrophic villi (40x, bar = 100 µm) and (**B**) neutrophils accumulated into the lumen of crypts of the ileum (white arrow) (400x, bar = 20 µm). Ileum from an uninfected calf with an intact epithelium (**C**) and crypt without any inflammatory cells (**D**). (**E–G**) Transcriptional gene expression of the *TLR4* (**E**), IL8, IL10 and TNFɑ (**F**), and β-defensins *TAP* and *LAP* and cathelicidin *BMAP28* (**G**) in the ileum of calves challenged by EHEC O157: H7. RT-qPCR expression of mRNA genes in each sample was conducted in triplicate and the mean + the standard error (SEM) shown. The expression of mRNA is relative to the uninfected controls. Only significant comparisons (*P* < 0.05) are noted (one-way ANOVA using Tukey’s post hoc test). A (*) denotes significant differences (p < 0.05) between one infected group and uninfected group and (#) denotes differences (p < 0.05) between infected groups (7 versus 14 d post-challenge). BMAP: bone marrow antimicrobial peptide. *TAP*: tracheal antimicrobial peptide. *LAP*: lingual antimicrobial peptide.

Ileum of calves infected with EHEC O157: H7 at 7 d post-challenge had inflammation with disrupted epithelium and atrophic villi (Fig. 2A), neutrophils present in the crypt lumen (Fig. 2B), and pronounced hyperplasia of B and T zones in Peyer ´s patches. Occasionally, layers of bacteria intimately attached to irregular epithelial surfaces and hemorrhages formed below the epithelium in infected calves. Uninfected calves showed no histological alterations in the ileal epithelium and crypts (Fig. 2C,D). At 14 d post-challenge, infected calves still showed diffuse atrophy and epithelial degeneration of ileal villi, often associated with congestion and prominent neutrophil infiltration.

In terms of innate effectors during EHEC O157: H7 infection, expression of *TLR4* mRNA did not change in the ileum of infected calves (Fig. 2E). Levels of *IL-8* mRNA were higher in infected calves at 7 d post-challenge compared to the levels found in uninfected calves and calves at 14 d post-challenge; no differences were observed between uninfected calves and calves at 14 d post-challenge (Fig. 2F). No change in gene expression was observed for *IL10* and *TNF* *α* between infected and uninfected calves (Fig. 2F). Transcriptional mRNA expression of β-defensin *TAP* was upregulated in the ileum of infected calves at 7 d post-challenge compared to the levels found in calves at 14 d post-challenge and in uninfected controls (Fig. 2G). *LAP* mRNA expression levels were similar between uninfected calves although *LAP* mRNA decreased in calves at 14 d post-challenge compared with uninfected calves (Fig. 2G). Cathelicidin *BMAP28* was similarly expressed in infected and uninfected calves (Fig. 2G).

### Colitis associated with the β-defensins synthesis in EHEC O157: H7 infected calves

**Figure 3:**
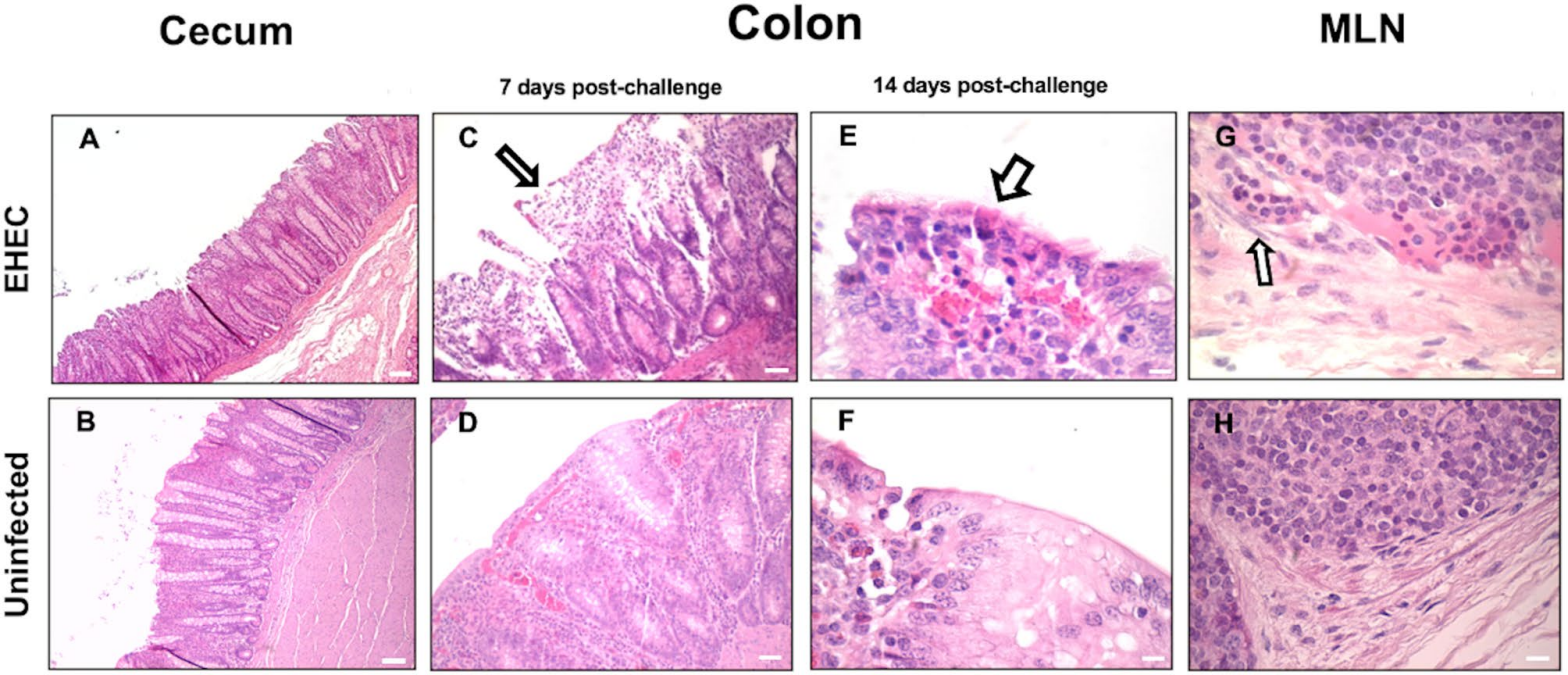
(**A-H**) Microphotographs (hematoxylin and eosin stain) of the cecum, colon, and mesenteric lymph node from calves infected by EHEC O157: H7 (EHEC) (**A, C, E, G**) and uninfected control calves (**B, D, F, H**). (**A**) Microphotograph of cecum from an infected calf with mild inflammation and disrupted epithelium and (**B**) from an uninfected calf (40x, bar = 100 µm). (**C**) Microphotograph of the colon with disrupted epithelium and subjacent cellular infiltrates (white arrow) at 7 d post-challenge (40x, bar = 100 µm) and (**D**) the corresponding uninfected control colon. (**E**) Microphotograph of colons of an EHEC O157: H7 infected calf at 14 d post-challenge with sub-epithelial congestion (400x, bar = 15 µm) and (**F**) from an infected calf. (**G**) Mesenteric lymph node with inflammatory cells infiltrating the subcapsular sinus (white arrow) in an EHEC O157: H7 infected calf at 7 d post-challenge and (**H**) mesenteric lymph node from a healthy uninfected calf (400x, bar = 15 µm).

Mild typhlitis was observed in infected calves at 7 d post-challenge with diffuse inflammatory cell infiltration in lamina propria of the cecum (Fig. 3A) while the cecum of uninfected calves showed no alterations (Fig. 3B). Colitis was also evident in EHEC O157: H7 infected calves, displaying disrupted epithelium in colons at 7 d post-challenge (Fig. 3C) while uninfected calves showed no alteration (Fig. 3D). Moreover, infected calves showed neutrophils infiltrated in the subepithelium of colons at 14 d post-challenge (Fig. 3E) while uninfected calves had no lesions (Fig. 3F). Mesenteric lymph nodes in EHEC O157: H7 infected calves presented moderate hyperplasia of B and T zones, edema, and inflammatory cells infiltrating the subcapsular sinus (Fig. 3G) compared with unaltered mesenteric lymph nodes in uninfected calves (Fig. 3H).

**Figure 4:**
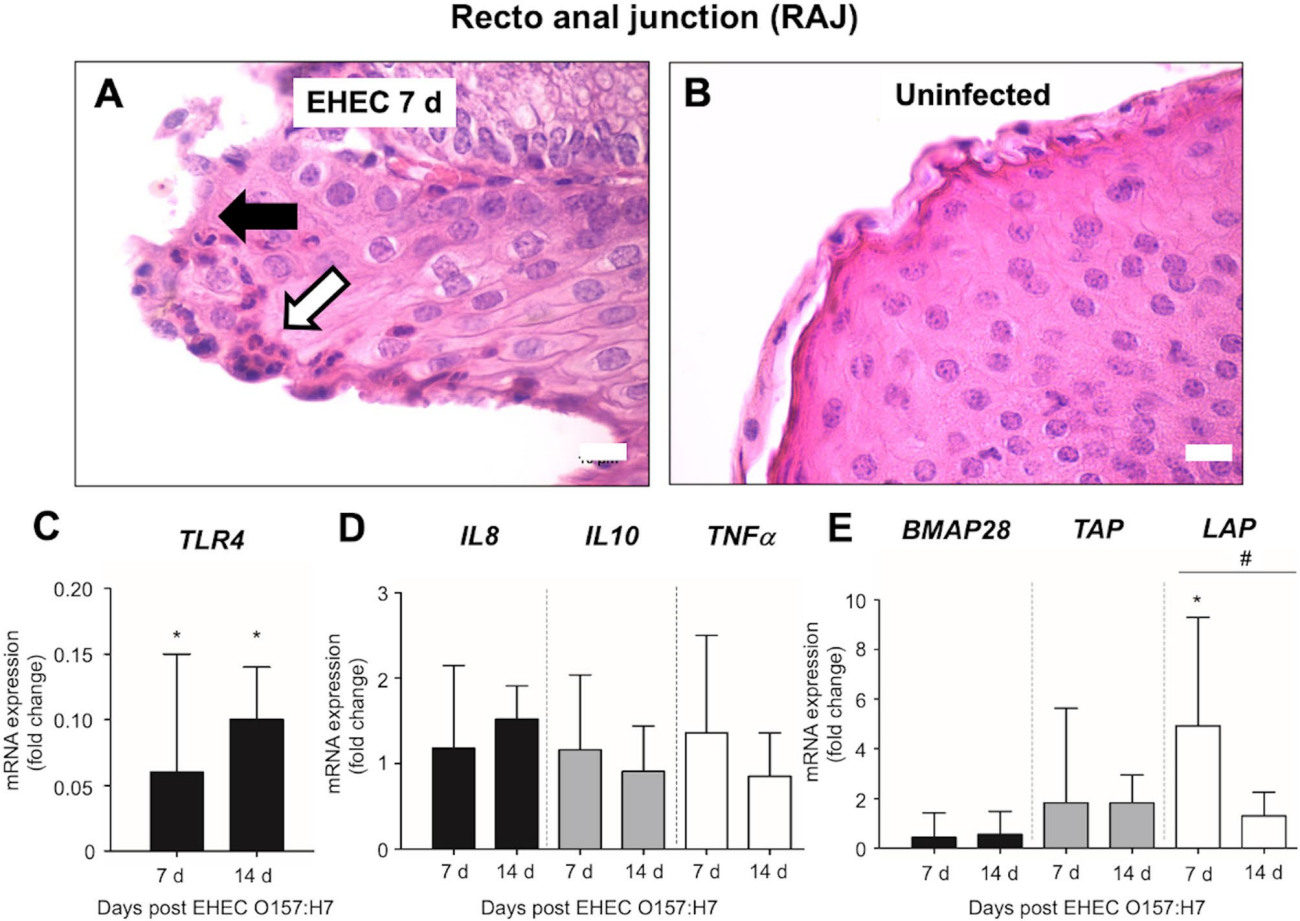
(**A-B**) Microphotographs (hematoxylin and eosin stain) of rectoanal junctions (RAJs) of (**A**) calves infected by EHEC O157: H7 (EHEC) 7 d post-challenge showing detached and sloughing epithelial cells (black arrow) and intraepithelial neutrophils (white arrow), and (**B**) uninfected calves with intact epithelium (400x, bar = 100 µm). Transcriptional gene expression of (**C**) *TLR4*, (**D**) *IL8, IL10* and *TNFɑ*, and (**E**) cathelicidin *BMAP28,* and β-defensins *TAP* and *LAP* in RAJs of calves challenged by EHEC O157: H7. RT-qPCR expression of mRNA genes in each sample was conducted in triplicate and the mean + the standard error (SEM) shown. The expression of mRNA is relative to the uninfected controls. Only significant comparisons (*P* < 0.05) are noted (one-way ANOVA using Tukey’s post hoc test). A (*) denotes significant differences (p < 0.05) between one infected group and uninfected group and (#) denotes differences (p < 0.05) between infected groups (7 versus 14 d post-challenge). BMAP: bone marrow antimicrobial peptide. *TAP*: tracheal antimicrobial peptide. *LAP*: lingual antimicrobial peptide.

Inflammation in EHEC O157: H7 infected calves extended until RAJs, where the epithelium was detached with sloughing epithelial cells and neutrophil microabscess appeared under the epithelium at 7 d post-challenge (Fig. 4A). Uninfected calves displayed an intact RAJ epithelium (Fig. 4B). Levels of *TLR4* mRNA in RAJ from infected calves at 7 and 14 d post-challenge were higher compared to the levels observed in uninfected calves, but with no difference between infected groups (7 versus 14 d) (Fig. 4C). Gene expression of *IL8, IL10,* and *TNF α* did not vary in RAJs of infected and uninfected calves (Fig. 4D). β-defensin *LAP* mRNA increased in RAJ of infected calves at 7 d post-challenge compared with uninfected calves and *LAP* mRNA levels decreased in infected calves at 14 d compared with 7 d post-challenge (Fig. 4E). Expression of cathelicidin *BMAP28* and β-defensin *TAP* did not differ in RAJs of infected and uninfected calves (Fig. 4E).

### Early depletion in goblet cell and mast cell populations in colons of EHEC O157: H7 infected calves

**Figure 5:**
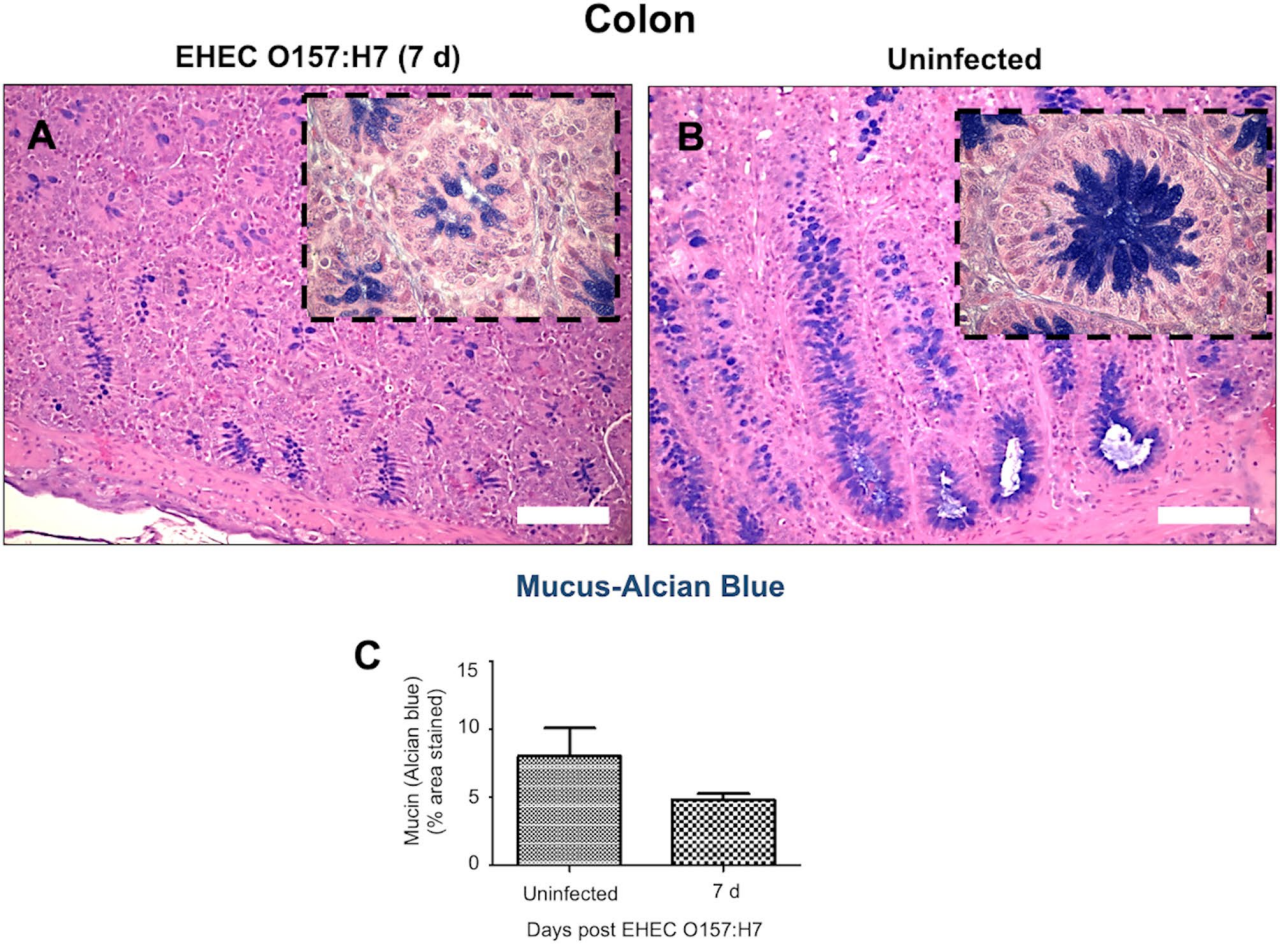
Microphotographs (alcian blue/hematoxylin stain) of goblet cells producing mucopolysaccharides and sialomucins (mucus) in (**A**) colons of calves challenged by EHEC O157: H7 (7 d post-challenge) and (**B**) colons of uninfected calves, and (**C**) their respective quantification. Bar = 100 µm.

Mucus producing goblet cells restricted to the bottom of the crypts in the colon of EHEC O157: H7 infected calves at 7 d post-challenge and they appeared in less number and poorly filled with mucus (Fig. 5A, Alcian blue) compared with uninfected colons (Fig. 5B) though the cell counting was not statistically different (Fig. 5C). Goblet cells in the cecum and RAJs were similarly present in number and distribution among the groups. (data not shown).

**Figure 6:**
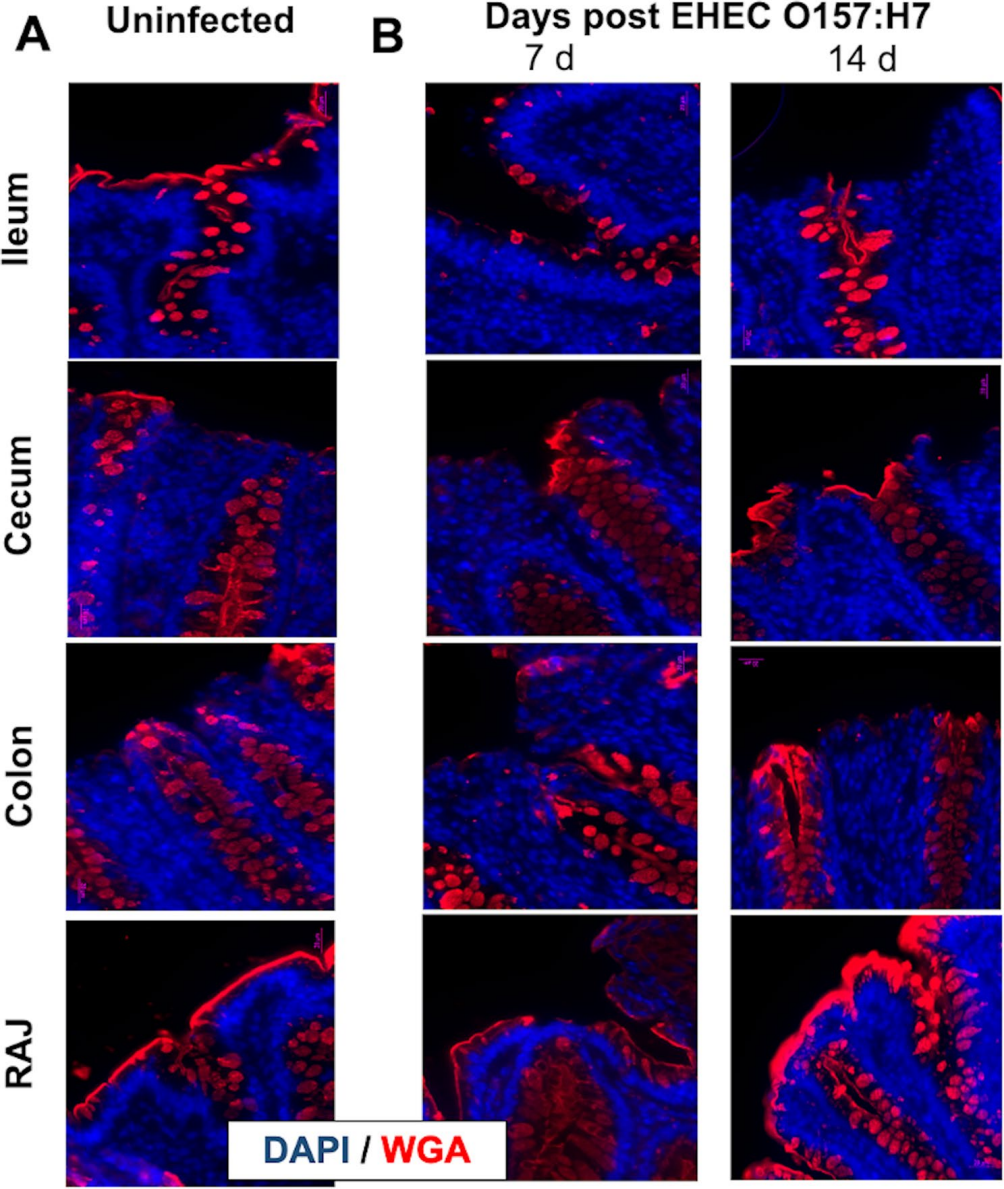
Microphotographs (lectin immunofluorescence with lectin wheat germ agglutinin; WGA) of cells producing N-acetyl-D-glucosamine and sialic acid (mucin) in the ileum, cecum, colon, and rectal anal junction (RAJ) of calves (**A**) uninfected and (**B**) challenged by EHEC O157: H7 (7 d and 14 d post-challenge). Infected small and large intestine displayed altered production of mucin glycoproteins with disparate production in the ileum, cecum, and colon and increased synthesis of mucin in RAJ at 14 d post-infection.

WGA lectin can bind oligosaccharides containing terminal N-acetylglucosamine (α-D-GlcNAc and NeuNAc) present in all cell membranes although these carbohydrates are abundantly expressed in mucus-producing goblet cells. These WGA^+^ glycans were abundant in the intestinal mucin layer of uninfected calves, showing filled round shape like- goblet cells and a continuous thin layer in ileum, cecum, colon, and RAJ (Fig. 6A). In EHEC O157: H7 infected calves, a discontinuous mucin layer pattern was observed where WGA lectin^+^ mucin containing goblet cells were dispersed in numbers and had a variable grade of filling across ileum, cecum, colon, and RAJ (Fig. 6B). In stark contrast, RAJs in calves at a later time of EHEC O157: H7 infection (14 d post-challenge) had exaggerated WGA mucin expression with filled goblet cells and a thick mucin layer (Fig. 6B).

**Figure 7:**
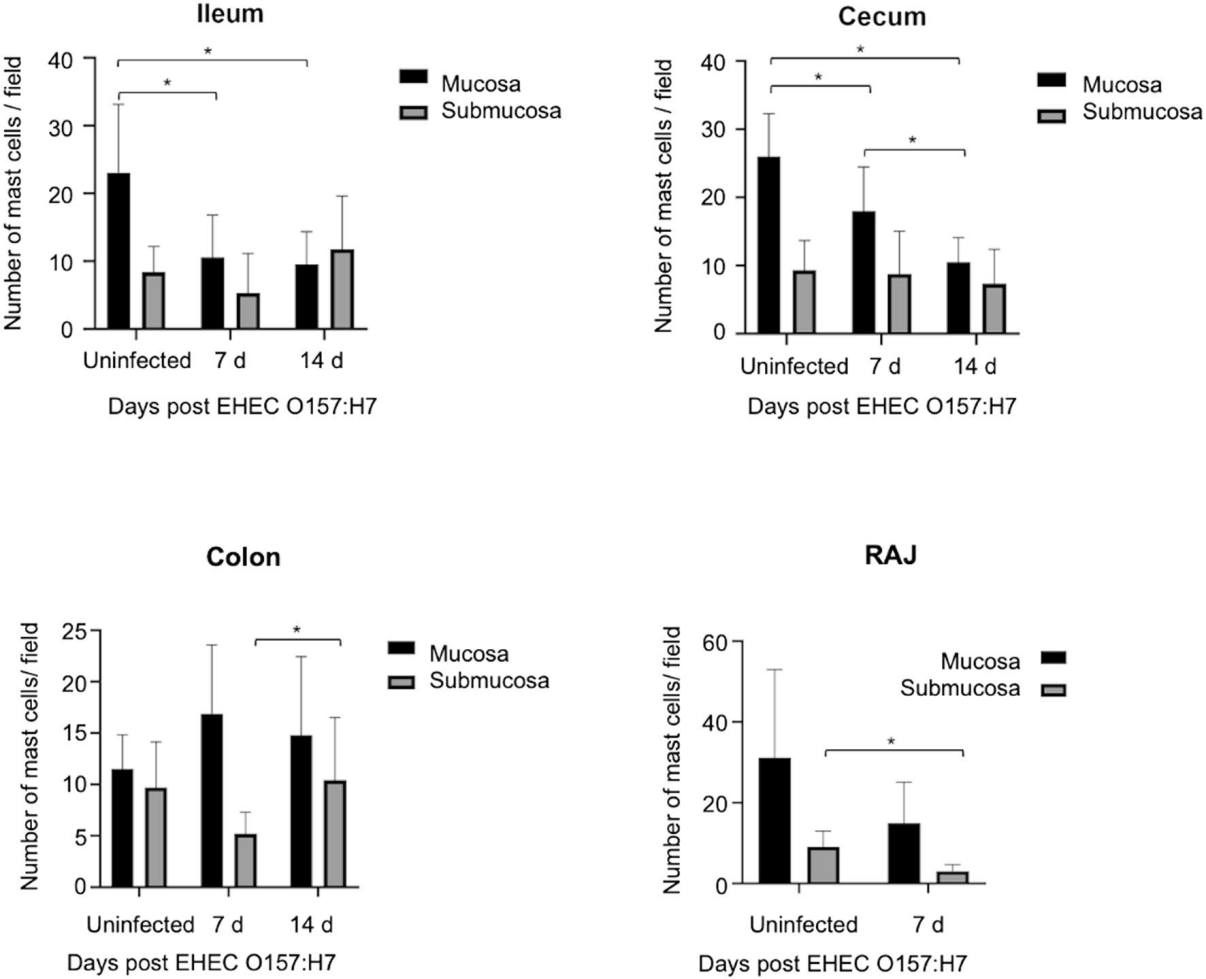
Quantification of mast cells in the mucosa and submucosa of ileum, cecum, colon, and rectal anal junction (RAJ) of calves challenged by EHEC O157: H7 (7 d and 14 d post-challenge). Mast cells were identified by toluidin blue staining (see Sup Fig. 1).

The number of mast cells reduced in ileal and cecal mucosal lamina propria of EHEC O157: H7 infected calves at 7 and 14 d post-challenge, although no difference was observed in the number of submucosal mast cells (Fig. 7, Sup Fig. 1). In RAJs, the number of submucosal mast cells decreased at 7 d post-challenge compared with the number of those cells in uninfected calves (Fig. 7, Sup Fig. 1). We observed the number of mast cells in the submucosa of colons in EHEC O157: H7 infected calves at 14 d post-challenge was higher compared with the number of those cells in calves infected at 7 post-challengee (Fig. 7, Sup Fig. 1).

### Analysis of diversity, richness and taxonomic composition of bacterial microbiota after EHEC O157: H7 infection

**Figure 8:**
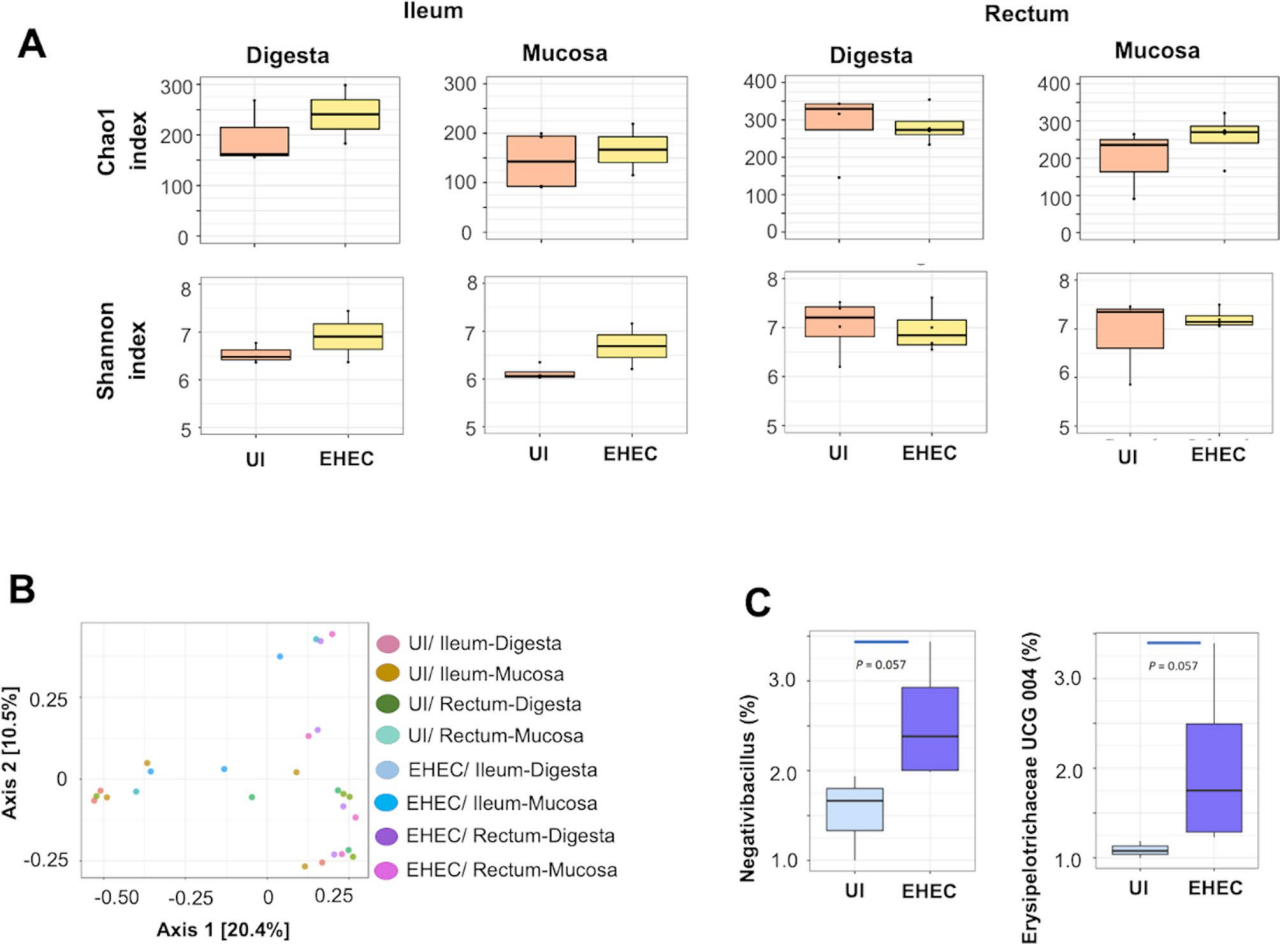
(**A**) Alpha diversity (Chao1 and Shannon index) in the ileum (mucosa-associated bacteria) and rectum (digesta and mucosa-associated bacteria) of calves uninfected (UI) or challenged by EHEC O157: H7 (EHEC). (**B**) Principle coordinate analysis (PCoA) based on Bray–Curtis distance in ileum and rectum (digest and mucosa-associated bacterial profiles) of calves uninfected (UI) or challenged by EHEC O157: H7 (EHEC). (**C**) The relative abundance of (*Negativibacillus* and *Erysipelotrichaceae* UCG 004 (%) in the rectum (mucosa-associated bacteria) of calves uninfected (UI) or challenged by EHEC O157: H7 (EHEC).

The sequencing of the bacterial 16S rRNA gene of all samples resulted in 908,059 total reads, with 33,631 ± 1,573 (average ± SE) reads per sample. After quality control and removal of potential contaminations, the remaining 228,703 reads were collapsed into 3,112 ASVs, with an average of 8,470 ± 457 reads and 206 ± 15 ASVs per sample, based on a 99% nucleotide sequence similarity. Both Chao1 and Shannon indexes were similar between uninfected and EHEC O157: H7 infected calves in ileum digesta, ileum mucosa, rectum digesta, and rectum mucosa at 14 d post-challenge (Fig. 8A, Sup Fig. 2 with rarefaction analysis). Similarly, principal coordinate analysis (PCoA) based on Bray–Curtis distance showed no cluster of digesta and mucosa-associated bacterial profiles between uninfected and EHEC O157: H7 infected groups in ileum and rectum, as indicated by the PERMANOVA analysis (*P* = 0.238) (Fig. 8B).

In terms of ileal bacterial composition, we revealed a total of 7/28/46, 7/15/26, 6/15/24, and 3/8/15 bacterial phyla/families/genera (relative abundance > 0.1% and present in more than half samples) in ileum mucosa (uninfected), ileum mucosa (EHEC O157: H7), ileum digesta (uninfected), and ileum digesta (EHEC O157: H7) associated bacteria, respectively (Sup Fig. 3). No difference was observed in the relative abundance of any phylum, family, or genus in the infected and uninfected calves in either ileum digesta or mucosa. We observed that *Firmicutes* was the predominant phylum in both ileum digesta (63.7%) and mucosa (74.0%). *Clostridiaceae* 1 (23.2%) and *Ruminococcaceae* (24.8%) were the predominant families in ileum mucosa and digesta, respectively. *Candidatus arthromitus* was the predominant mucosa-associated bacteria, representing 13.7% and 28.2% of total bacterial genera in uninfected and EHEC O157: H7 infected groups, respectively. The second most abundant genus was *Escherichia-Shigella* (*Enterobacteriaceae* family) in uninfected calves (9.84%) and *Eimeria praecox* (*Cyanobacteria* family) in infected calves (8.13%) (Table 1). *Romboutsia* (17.5%) and *Escherichia-Shigella* (11.7%), as well as *Turicibacter* (9.9%) and *Escherichia-Shigella* (9.2%), were the predominant bacterial genera in digesta-associated bacteria in uninfected and EHEC O157: H7 infected group, respectively (Table 1).

**Table 1:** Taxonomic composition of most relative abundant bacteria in ileum mucosa, ileum digesta, rectum mucosa, and rectum digesta (4 locations) from uninfected and EHEC O157: H7 infected groups (R: relative abundance %, expressed as mean ± standard deviation).

UNINFECTED				EHEC O157: H7			
Phylum	Family	Genus	R	Phylum	Family	Genus	R
**Ileum mucosa**							
Firmicutes	Clostridiaceae	*Candidatus arthromitus*	13.7 ± 16.4	Firmicutes	Clostridiaceae	*Candidatus arthromitus*	28.2 ± 41.9
Proteobacteria	Enterobacteriaceae	*Escherichia-Shigella*	9.84 ± 16.9	Cyanobacteria	Eimeria praecox	*Eimeria praecox*	8.13 ± 8.64
**Ileum digesta**							
Firmicutes	Peptostreptococcaceae	*Romboutsia*	17.5 ± 15.2	Firmicutes	Erysipelotrichaceae	*Turicibacter*	9.99 ± 13.6
Proteobacteria	Enterobacteriaceae	*Escherichia-Shigella*	11.7 ± 15.2	Proteobacteria	Enterobacteriaceae	*Escherichia-Shigella*	9.22 ± 4.30
**Rectum mucosa**							
Firmicutes	Lachnospiraceae	NA	12.8 ± 12.0	Firmicutes	Ruminococcaceae	*Ruminococcaceae* UCG-005	19.2 ± 12.9
Firmicutes	Ruminococcaceae	*Ruminococcaceae* UCG-005	12.8 ± 11.3	Firmicutes	Lachnospiraceae	NA	13.8 ± 4.17
**Rectum digesta**							
Firmicutes	Ruminococcaceae	*Ruminococcaceae* UCG-005	17.0 ± 12.8	Firmicutes	Ruminococcaceae	*Ruminococcaceae* UCG-005	23.6 ± 15.0
Firmicutes	Lachnospiraceae	NA	11.7 ± 5.23	Firmicutes	Lachnospiraceae	NA	12.9 ± 3.59

In the rectum, a total of 7/19/39, 6/21/52, 5/20/49, and 5/20/48 bacterial phyla/families/genera (relative abundance > 0.1% and present in more than half samples) were observed in rectum mucosa (uninfected), rectum mucosa (EHEC O157: H7), rectum digesta (uninfected), and rectum digesta (EHEC O157: H7) associated bacteria, respectively (Sup Fig. 3). No difference was observed in the relative abundance of any phylum or family in the infected and uninfected calves in either ileum digesta or mucosa. *Firmicutes* (63.3% and 72.5%) was the predominant bacterial phylum, and *Ruminococcaceae* (32.4% and 41.4%) was the predominant bacterial family in both rectal mucosa and digesta, respectively. Unclassified *Lachnospiraceae* (12.8%) and *Ruminococcaceae* UCG-005 (12.8%) were the predominant genera in mucosa-associated bacteria in the uninfected group. *Ruminococcaceae* UCG-005 was also the predominant bacterial genus in rectal mucosa-associated bacteria (EHEC O157: H7, 19.2%), digesta associated bacteria (uninfected, 17.0%), and digesta associated bacteria (EHEC O157: H7, 23.6%), respectively (Table 1). The genus *Escherichia*-*Shigella* belonging to the family *Enterobacteriaceae* was only detected in the rectal digesta of the EHEC O157: H7 group (0.33%), but not in the uninfected group. The mean value of the relative abundance of *Negativibacillus* (P = 0.057; Fig. 8C) and *Erysipelotrichaceae* UCG 004 (P = 0.057; Fig. 8C) in rectum mucosa was higher but not statistically significant in EHEC O157: H7 infected calves at 14 d post-challenge compared to uninfected calves.

## Discussion

This study describes modifications in the gut innate immune defenses, including β-defensins *TAP* and *LAP*, *IL8*, and *TLR4* expression, the mucus layer, and the number of mast cells, in calves infected with and shedding EHEC O157: H7. The use of an EHEC O157: H7 (438/99) strain containing enterohemolysin, γ-intimin, T3SS, Stx, and pO157 plasmid virulence factors may have contributed to its colonization^36–40^ and the innate immune hallmarks, including the increased transcription of *IL8* in the ileum and *TLR4* in RAJs in infected calves in the early infection (7 d post-challenge). For instance, EHEC O157: H7 lacking Stx (*Stx*^*−*^) developed lower inflammatory responses with decreased IL6 and IL8 release in RAJ and Peyer´s patches in infected calves^41^. Likewise, H7 flagellin^10,42^, and long polar fimbriae^43^ and FliC flagellin^44^ induced IL-8 expression, while LPS from EHEC O157: H7 is an inducer of TLR4^42,45^. The intestinal epithelium could be particularly key in these initial gut responses; it produces early *IL8* via transcription factor NF-kβ (Berin et al. 2002) and *TLR4* is highly expressed in the distal colon^46^. *E. coli* signaling into intestinal epithelium through TLR4 is likely activating MAP kinase (p38-ERK 1/2)/NF-kβ and producing IL8 (Berin et al. 2002). CXCL-8/IL8 chemo attracts neutrophils to the gut and eventually across the epithelial layer towards the intestinal lumen^47,48^. Thus, the early expression of *IL8* in the gut of EHEC O157: H7 infected calves might contribute to the accumulations of neutrophils in the lamina propria and lumen observed previously^32^ and in our study.

The intestinal mucus layer is responsible for preventing *E. coli* adherence to the epithelial cells and the formation of attaching/effacing lesions. We observed that EHEC O157: H7 disrupted the intestinal mucin barrier during early infection (7 d post-challenge); a mechanism that is likely favoring EHEC O157: H7 colonization. An impaired mucin layer could affect sentinel goblet cells, which sense LPS from invading pathogens and stimulate goblet cells in the crypts to secrete MUC2 via surface TLR4 and downstream NOD-like receptor family pyrin domain containing 6 (Nlrp6) inflammasome activation^49^. Such alterations of the mucin layers could be attributed to metalloproteases (StcE) produced by EHEC, which cleave mucin-type glycoproteins^50^, reduce MUC2 levels in goblet like (LS174T) cells and increase bacterial binding and pedestal formations^51^.

We detected a later increase of WGA lectin (that binds N-acetyl-D-glucosamine and sialic acid) in infected RAJs (14 d post-challenge). RAJ is the preferred site of EHEC O157: H7 colonization in cattle^52^ and this mucin production may be an attempt by the host to eliminate the pathogen. N-acetylglucosamine and N-acetylneuraminic acid sugars derived from mucin can inhibit EHEC adhesion to epithelial cells^53^. Moreover, in a host counter-attack, pro-inflammatory cytokine/chemokines (TNFα, IL8) enhanced mucin MUC2 production and reduced adhesion of EHEC O157: H7 in colonic epithelial (HT-29) cells and in cattle colonic explants (Xue et al. 2014). Taken together, EHEC O157: H7 may first reduce the mucus synthesis in ileum and cecum as denoted by the discontinued mucin layer and less full goblet cells. An ongoing inflammation with IL8 synthesis and TLR4 activation could promote mucin production and re-establish the mucin layer, mostly in areas where the bacteria inhabit, such as RAJs. However, the relationship between mucin glycosylation and susceptibility to *E. coli* is complex. For instance, *O*-acetylated sialic acids (e.g., neuraminic acid Neu5Ac) linked to glycan chains of mucin can be used by EHEC as carbon sources^54^. Further studies with multiple lectins could decipher the mucin glycan dysregulation during EHEC O157: H7 infection.

The role of host defense peptides is of particular interest in cattle, which are rich in cathelicidins and β-defensin^13,55^. We determined increased transcription of β-defensin *TAP* in ileum and *LAP* in RAJs of calves infected with EHEC O157: H7. The prevalence of LAP in bovine gut defenses agrees with studies showing *LAP* mRNA expression all along the digestive tract in calves^25^. An early synthesis of β-defensins could contribute to the neutrophil responses observed in the gut of EHEC O157: H7 infected calves. Previous studies in monogastric animal species showed β-defensins suppressed neutrophil apoptosis^56^, thus extending neutrophil life span. Additionally, β-defensins may aid in recruiting neutrophils to injury sites. Human β-defensins 2/3 and their mouse orthologues (β-defensins 4/14), interacted with CCR2, a chemokine receptor expressed on monocytes, macrophages, and neutrophils^57^. On the other hand, *BMAP28*, a key bovine cathelicidin in mammary epithelial cells^58^, was unaltered during EHEC O157: H7 infection. Since BMAP28 is mostly derived from myeloid leukocytes, this cathelicidin could appear later when leukocytes arrive and massively infiltrate the gut. In that case, cathelicidins could promote further neutrophil influx to injury sites; as cathelicidin was chemoattractant in the skin^12^ and induced the expression of CXCL8 in keratinocytes^59^ and colonocytes^60^.

Intestinal mast cells are particularly responsive to *E. coli*^61^ and its virulent factors (e.g., α-hemolysin)^62^. Indeed, mast cells conferred protection in mice with colitis caused by the related attaching/effacing enterobacteria, *Citrobacter rodentium*, reducing the bacterial load, and preventing dissemination^63^. We showed that the number of mast cells decreased in the lamina propria of ileum and cecum but increased in the RAJ submucosa in calves infected with EHEC O157: H7. Mast cells in the lamina propria predominantly contain high levels of tryptase^64^ whereas submucosal mast cells are rich in tryptase, chymase, and carboxypeptidase^30,65^. The increased number of submucosa mast cells in RAJs (the most active site in terms of infection) infers the release of multiple pre-formed products, including leukotrienes^66^, chemokines CXCL1/2^67^ and a variety of proteases. Mast cells and their products could also promote neutrophil infiltration as they showed affinity to FimH-expressing *E. coli*^68^ and killed microbes by producing antimicrobial cathelicidins^69^. Thus, mast cell defense is afflicted and/or exhausted at the mucosa during early EHEC O157: H7 infection but submucosal mast cells in the colon could fight infection after its onset.

The intestinal microbiota is expected to reflect the pathogen colonization or furthermore, protect from EHEC O157: H7 infection. Commensal bacteria that degrade mucin can change the concentration of *O*-glycans affecting the colonization of EHEC^53^. We observed no differences in ileum digesta or mucosa-associated bacterial genera between uninfected and EHEC O157: H7 infected (14 d post-challenge) calves although the latter had a lower number of bacterial genera for both ileum mucosa (26 vs. 46) and digesta associated (15 vs. 24) microbiota. Specifically, the ileum mucosa of infected calves lacked *Butyrivibrio*^70^ and *Roseburia*^71^, two butyrate-producing genera beneficial in the early life of calves. On the other hand, EHEC O157: H7 infected calves lacked *Turicibacter* in the ileum mucosa, a genus commonly found in the intestines of humans and animals^72^ and increased in feces of mice infected with *E*. *coli*^73^. These differences in the ileal microbiota indicate an impaired ability of infected calves to maintain homeostasis. In the rectum, a higher relative abundance of *Negativibacillus* and *Erysipelotrichaceae* UCG-004 genera were observed in mucosa-associated bacteria of EHEC O157: H7 infected calves; presumptive of a distal gut dysbiosis. *Negativibacillus* genus was higher in cecal contents of mice with obesity-related disorders^74^ and *Erysipelotrichaceae* UCG-004 prevailed in feces of piglets with diarrhea^75^. On the other hand, the *Erysipelotrichaceae* family was reported to be involved in IgA production in humans^76^ and it could contribute to the immune response against EHEC in cattle. In comparison with previous studies, we did not observe abundant *Campylobacter* spp. and *Sutterella* spp.^77^, likely due to individual and environment particularities. Of note, despite high dose infective inoculum (10^10^ CFU), the abundance of the *Escherichia-Shigella* taxon did not increase in challenged calves; a phenomenon that was previously reported^77,78^.

In summary, we defined innate immune responses in the early pathogenesis of EHEC O157: H7 in cattle. Those innate hallmarks included an altered gut mucin layer and depletion of mast cells in conjunction with an activated innate response in RAJs, perhaps due to higher bacterial colonization. Moreover, we showed increased gene expression of β-defensins *TAP* and *LAP* in the gut of calves against EHEC O157: H7, that together with chemoattractant *IL8,* likely promote the arrival of leukocytes. Such information on innate mucosal defenses aids in understanding survival mechanisms of EHEC O157: H7 in the reservoir and are potential therapeutic targets for controlling infectious diseases in livestock.

## Methods

### Ethics statement

All the experimental protocol for using animals in the study was approved by the ethics committees along with the University of Calgary Animal Care Committee (AC18-0034) and the Institutional Animal Care and Use of Experimentation Animals Committee (CICUAE) of Instituto de Agrobiotecnología y Biología Molecular IABIMO, INTA-CONICET and was followed according to the Canadian Guidelines for Animal Welfare (CGAW).

### Enterohemorrhagic *Escherichia coli* (EHEC) O157: H7

EHEC O157: H7 bacteria (strain 438/99; enterohemolysin, *γ*-intimin, EspA, EspB, Stx2c, pO157 plasmid positive and nalidixic acid-resistant) was isolated from a healthy cow and used in previous studies^79^. For infecting calves, EHEC O157: H7 was cultivated in Luria–Bertani (LB) broth containing nalidixic acid antibiotic (20 µg/mL, Sigma) (200 rpm, 18 h, 37 °C). Overnight cultures were diluted (1/30) in LB broth with nalidixic acid (20 µg/mL) and grown until 1.1 OD_600nm_ (5 h), centrifuged (6,000 rpm, 4 °C; 5 min) and the bacterial pellet was resuspended in sterile phosphate-buffered saline (PBS). Calves were challenged with an inoculum containing 10^10^ CFU in 10 mL. The amount and viability of CFU were confirmed by plating on serial dilutions on Sorbitol MacConkey agar (Oxoid) containing nalidixic acid (20 µg/mL), potassium tellurite (2.5 µg/mL), and cefixime (0.05 µg/mL) (CT-SMAC-NAL)^80^.

### Experimental EHEC O157: H7 infection in cattle

Male (70 d-old) Holstein Friesian calves (n = 13) with similar weight and free of STEC (as determined by enrichment of recto-anal mucosal swabs streaked onto sorbitol Mac Conkey agar) were selected from a local dairy farm (Buenos Aires, Argentina). At the farm, neonatal calves stayed with their dams for 3 d and drank colostrum directly from them. Then, calves were weaned and consumed bottled colostrum (two daily intakes) for up to 1 wk. After this first wk of age, calves received commercial milk replacer (twice a day), containing powdered milk, corn, soy, fiber and minerals, and vitamins. When calves were 70 d-old, they were transported and housed in biosafety level 2 (BSL2) pens where they received the same diet during the first 2 d of acclimatization and ivermectin (1%, Ivomec Merck) to control intestinal nematodes. During the experiment, calves fed increasing amounts of alfalfa pellets for up to 50% of the diet with ad libitum access to hay and water. Calves were orally inoculated into the rumen with EHEC O157: H7 (10^10^ colony-forming unit, CFU, in sterile phosphate-buffered saline, PBS) using an esophageal tube feeder and euthanized at 7 d (n = 4; group 1) or 14 d post-challenge (n = 4; group 2). The remaining calves (n = 5 calves, group 3) received orally inert buffer only and were euthanized 14 d post-challenge.

Clinical signs and fecal consistency were monitored twice every day in each animal. At the necropsy, tissue Sects. (5 cm^2^) were systemically taken in each calf from the same anatomical sites of the ileum, cecum, colon, RAJ, and mesenteric lymph nodes. Each tissue sample, aseptically and individually collected, was rinsed with PBS, cut into 3 portions (1 × 2 cm each), and placed for bacterial isolation in trypticase soy broth (TSB, Oxoid), histopathology in 10% neutral buffered formalin, and gene expression analysis in RNA*later* stabilization solution (AM7021; Thermo Fisher).

### EHEC O157: H7 shedding in calves

The burden of EHEC O157: H7 was quantified in RAJ swabs at 1, 6, and 14 d post-challenge. Swabs were vortexed in TSB and plated on serial dilutions on Sorbitol MacConkey agar (Oxoid) containing nalidixic acid (20 µg/mL), potassium tellurite (2.5 µg/mL), and cefixime (0.05 µg/mL) (CT-SMAC-NAL). When direct cultures were negative, swabs were enriched (37 °C, 18 h) and an aliquot (1 mL) was subjected to *E. coli* O157 immunomagnetic separation (IMS, Dynabeads anti-*E. coli* O157, Invitrogen Dynal AS) before platting them on CT-SMAC-NAL. Culture-positive samples by IMS were considered positive (value of 10 CFU) whereas culture-negative samples by IMS were deemed negative (value of 1 CFU). Non-sorbitol-fermenting colonies were tested for *E. coli* O157 lipopolysaccharide (LPS) by latex agglutination (Oxoid) and confirmed by multiplex PCR for the *stx1, stx2, eae,* and *rbf*_*O157*_ genes^35,81^. RAJ segments obtained from terminated calves were immediately enriched on TSB (overnight), aliquoted (3 mL), and subjected to IMS as described for *E. coli* O157: H7 determination.

### Histological and lectin histochemistry

Formalin-fixed, paraffin-embedded sections were cut (7 μm) and stained using hematoxylin and eosin (H&E) or toluidine blue for histological examination.

The mucin layer and goblet cells in the intestines were characterized using Alcian blue (Periodic acid-Schiff; PAS) and highly glycosylated proteins in mucin were labeled with lectin^82–84^. For lectin histochemistry, paraffin wax-embedded Sects. (5 μm) were dewaxed and treated with hydrogen peroxide (0.3% in methanol, 30 min, room temperature) to inhibit endogenous peroxidase. Sections were rinsed several times in PBS (0.01 M, pH 7.2), blocked with bovine serum albumin (0.1% in PBS, 15 min) and incubated with biotinylated WGA lectins (*Triticum vulgaris* Lectin Kit BK 1000; Vector,) specific α-D-GlcNAc and NeuNAc highly present in the colonic mucin (30 μg/mL in PBS, 1 h, room temperature). Slides were incubated with avidin–biotin-peroxidase complex (ABC) (Vector; 45 min) and horseradish peroxidase activated by a diaminobenzidine commercial kit (Dak, 1–2 min). Specimens were rinsed in distilled water, dehydrated with graded ethanol solutions, cleared in xylene, and mounted in Permount (Fisher Scientific).

Images of gut sections were captured using a digital video camera (Nikon Y-TV 55) attached to a microscope (Nikon Eclipse E200). Captured images (TIFF format) were analyzed using an image analyzer (Rasband, W.S., ImageJ; https://imagej.nih.gov/ij/, 1997–2018). For calculating the mucin area (positive for Alcian blue), digital photographs of 3 randomly selected areas were subjected to thresholding detection and automatic measurement to avoid the inclusion of empty areas into the measured area. The color picture was converted to 8-bit greyscale and the threshold was manually adjusted to detect any region containing tissue sections (grey to black) and exclude the empty areas (white). The area of interest was automatically measured by the software and the percentage of the stained area compared among groups. Mast cells stained by toluidine blue were quantified by manual counting of labeled cells at 40 × magnifications in at least 10 field-digitalized images^85^. For the quantifying analysis (H&E, counting mast cells), slides were examined independently by two veterinary pathologists and scored blindly^86^.

### Transcriptional expression of TLRs, pro/anti-inflammatory cytokine, and host defense peptides in the bovine intestine

Relative messenger RNA (mRNA) level of *TLR4*, cytokines *IL8*, *IL10* and *TNFα*, cathelicidin *BMAP28*, and *β*-defensins *LAP* and *TAP* were determined in the ileum and RAJ by real-time reverse transcription-quantitative polymerase chain reaction (RT-qPCR). Total cellular RNA was extracted using TRIzol (Invitrogen, Thermo Fisher), followed by polytron tissue homogenization^87^. Each sample was treated twice with chloroform and centrifuged (10 min, 12,000×*g*, 4 °C). Nucleic acids were precipitated by isopropanol and incubated (overnight, − 80 °C). The pellets were then washed with ice-cold 70% ethanol and resuspended in RNAse-free water. RNA samples were treated with DNAse (DNAseI Ambion, Thermo Fisher Scientific) and 1 volume of LiCl (10 M). Complementary DNA (cDNA) was obtained by SuperScript II reverse transcriptase (Thermo Fisher Scientific). qPCR reactions were carried out with Taq Platinum DNA polymerase (Invitrogen) and SYBR reagent (Thermo Fisher) and performed using standard cycling conditions (Applied Biosystems StepOne plus SDS). *Glyceraldehyde-3-phosphate dehydrogenase* (*GAPD*) was used as the housekeeping gene^88^ after confirming its gene expression stability in control bovine tissues and correlation with other housekeeping genes *(β-actin* and *UBQ*). All primer sequences are listed in Table 2 and checked for efficiency. Negative controls for cDNA synthesis and PCR procedures were included in all cases. Each reaction was performed in duplicate and the target mRNA values were corrected relative to *GAPDH*. The generated qPCR curves were analyzed using LinReg tool^89^ and ratio calculation and statistical analysis with Fg software^90^. The results were reported as mean fold changes of target gene transcription levels in infected vs. uninfected (control) groups or at 7 d vs 14 d post-infection.

**Table 2:** Bovine primers used for mRNA relative quantification (qRT-PCR).

Gene	Primer sequence	Accession #	Annealing temp (°C)	Reference
IL8	F: GTTGCTCTCTTGGCAGCTTT R: GGTGGAAAGGTGTGGAATGT	NM_173925.2	60	^95^
IL10	F: TGTATCCACTTGCCAACCAG R: CAGCAGAGACTGGGTCAACA	NM_174088.1	60	^95^
TLR4	F: AACCACCTCTCCACCTTGATACTG R: CCAGCCAGACCTTGAATACAGG	XM_019966825.1	60	^96^
GAPDH	F: GGGTCATCATCTCTGACCT R: GGTCATAAGTCCCTCCACGA	NM_001034034.1	60	^95^
CATH5 (BMAP28)	F: TGCTGAAAGAGTGTGTGGGG R: GGCCCACAATTCACCCAATTC	XM_024982399.1	60	Primer-BLAST (NIH)^97^
LAP	F: ACAGATTGGCACCTGTCTCG R: CTCTGTCCAAGGGCACAGTT	NM_203435.4	60	^98^
TAP	F: TCTTCCTGGTCTGTCTGCT R: GCTGTGTCTTGGCCTTCTTT	NM_174776.1	60	^99^
TNFα	F: GCCCTCTGGTTCAGACACTC R: AGATGAGGTAAAGCCCGTCA	XM_027524121.1	60	Primer-BLAST (NIH)^97^

### Gut microbiota profiling using amplicon sequencing

Mucosal tissue and digesta samples were collected from the ileum and rectum of uninfected and *E. coli* O157: H7 infected calves (14 d post-challenge). Mucosal tissue samples were rinsed with sterile PBS (pH 7.0) to remove the digesta and immediately snap-frozen. Samples of ileum and rectums were physically disrupted by a bead-beating step (FastPrep-24, MP Biomedicals). Total DNA was extracted from mucosal tissue and digesta samples of each gut region (220 mg wet weight) (QIAMP DNA stool kit, Qiagen), quantified (Qubit fluorometer, Qiagen) and stored (−80 °C) until further processing. The V3-V4 hypervariable regions of the 16S rRNA gene were amplified from the extracted DNA using universal primers 515F (50-GTGCCAGCMGCCGCGGTAA-30)^91^ and 806R (50-GGACTACHVGGGTWTCTAAT-30)^92^. Sequencing was performed on an Illumina MiSeq (2 × 300 bp) and sequence data were analyzed using Quantitative Insight into Microbial Ecology 2 (QIIME2) platform (version 2019.7; Bolyen et al., 2019). Paired sequences were demultiplexed with ‘demux’ plugin before subjecting to quality control using the ‘dada2′ plugin^93^. Dada2-based denoising identified features as amplicon sequence variants (ASVs). Taxonomy was assigned to ASVs using a pre-trained QIIME2-compatible SILVA database (released in July 2019, available at https://docs.qiime2.org/2019.7/data-resources/) with 99% identity for bacteria. Alpha diversity indexes, including Chao1 and Shannon indexes, were calculated with a sample depth of 4875 using QiIIME2 ‘diversity’ plugin. Principal coordinate analysis (PCoA) of the bacterial profiles based on Bray–Curtis distance was conducted using MicrobiomeAnalyst (https://www.microbiomeanalyst.ca)^94^. Permutational analysis of variance (PERMANOVA) was used to compare the difference in bacterial profiles between treatments. Identified nucleotide sequence accession numbers were deposited in the NCBI Sequence Read Archive (# SRR11547334- SRR11547362).

### Statistical analyses

Normality was assessed using D’Agostino & Pearson omnibus normality or Shapiro–Wilk (Royston) tests. Normally distributed (parametric) results are graphed as means, and bars represent standard errors (SEM) of the mean. All comparisons were performed by one-way ANOVA using Tukey’s post hoc test with Graph Pad Prism (5.0). For non-parametric microbial results, Wilcoxon signed-rank test was used to compare the difference in the relative abundance of bacterial genus between infected and uninfected calves using R (version 3.6.1). *P* values < 0.05 were considered statistically significant whereas 0.05 < *P* ≤ 0.10 as tendency.

## Supplementary information

Supplementary Information 1.
