# Supplementary material for: Early immune innate hallmarks and microbiome changes across the gut during Escherichia coli O157: H7 infection in cattle
Source: Sci Rep. 2020 Dec 9;10:21535. doi: 10.1038/s41598-020-78752-x (PMC7726576; doi:10.1038/s41598-020-78752-x)
Supplement: Supplementary file 1 — Supplementary Information 1. [file 41598_2020_78752_MOESM1_ESM.pdf]

Supplementary Figures

Early immune innate hallmarks and microbiome changes across the gut during *Escherichia coli* O157:H7 infection in cattle

Mariano Larzábal[1#], Wanderson Marques Da Silva[1#], Anmol Multani[2], Lucas E. Vagnoni[1], Dadin P. Moore[3], Maia S. Marin[3], Nahuel A. Riviere[1], Fernando O. Delgado[4], Daniel A. Vilte[4], Matias Romero Victorica[1], Tao Ma[5,6], Le Luo Guan[5], Paola Talia[1], Angel Cataldi[1], and Eduardo R. Cobo[2*]

[1] Agrobiotechnology and Molecular Biology Institute (IABIMO)-CICVyA. National Agricultural Technology Institute (INTA), National Scientific and Technical Research Council (CONICET), Hurlingham, Argentina.

[2] Production Animal Health, Faculty Veterinary Medicine, University of Calgary, Calgary, Canada.

[3] National Scientific and Technical Research Council (CONICET), National Agricultural Technology Institute (INTA), EEA-Balcarce, Balcarce Argentina.

[4] Veterinary Pathobiology Institute (IPVet) CICVyA, National Scientific and Technical Research Council (CONICET), National Agricultural Technology Institute (INTA), Argentina.

[5] Department of Agricultural, Food and Nutritional Science, University of Alberta, Edmonton, Canada.

[6] Feed Research Institute/Key Laboratory of Feed Biotechnology of the Ministry of Agriculture and Rural Affairs, Chinese Academy of Agricultural Sciences, Beijing, China.

Supplementary Figure 1. Representative histologic images of mast cells producing acidic polysaccharides components (purple) in ileum, colon and rectal anal junction (RAJ) of calves challenged by EHEC O157:H7 (7 d and 14 d post-challenge). Histological detection by toluidin blue staining. Bar = 50 μm.

Sup Fig 1

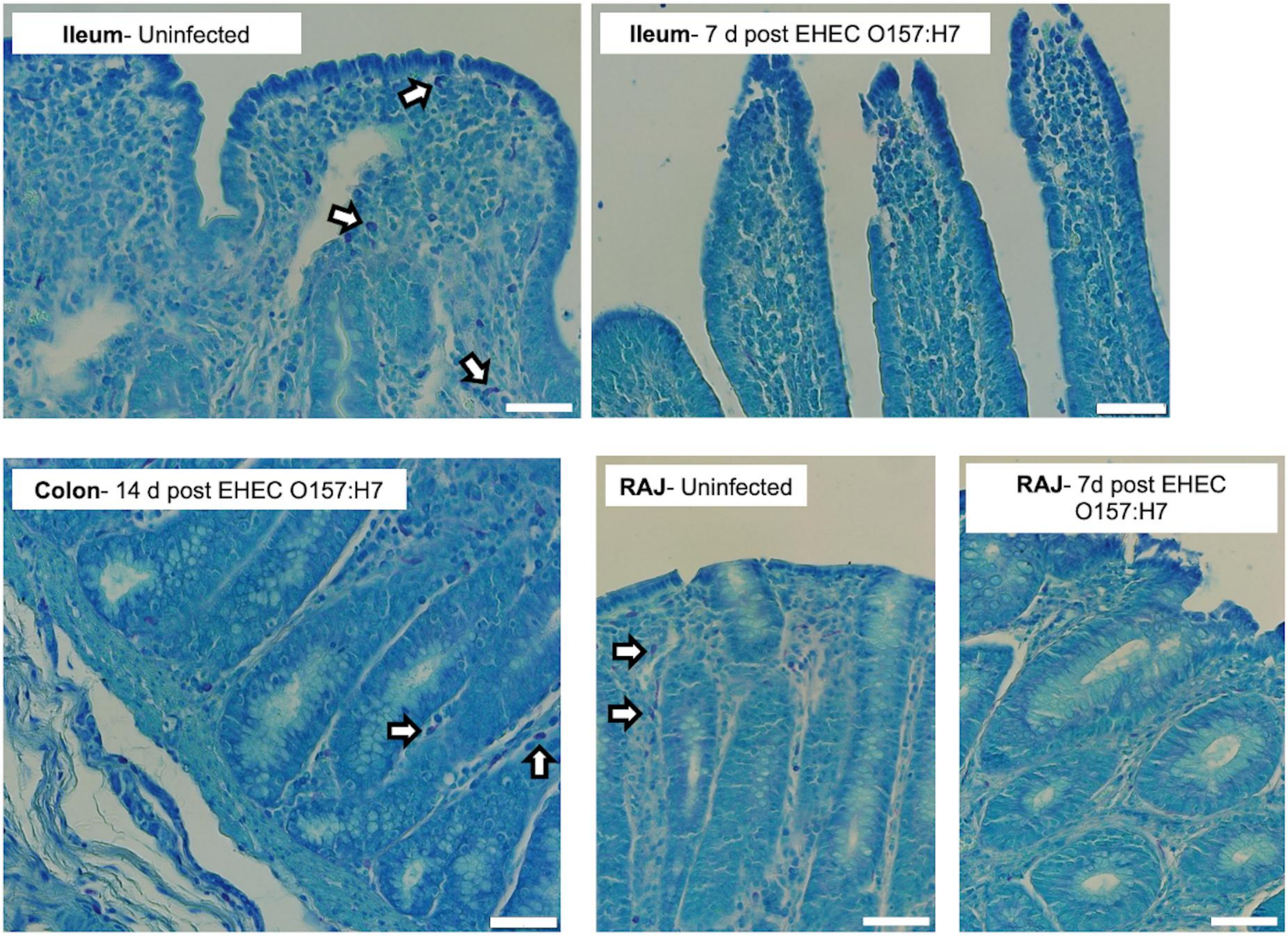

Ileum- Uninfected
Ileum- 7 d post EHEC O157:H7
Colon- 14 d post EHEC O157:H7
RAJ- Uninfected
RAJ- 7d post EHEC O157:H7

Supplementary Figure 2. Chao and Shannon index rarefaction

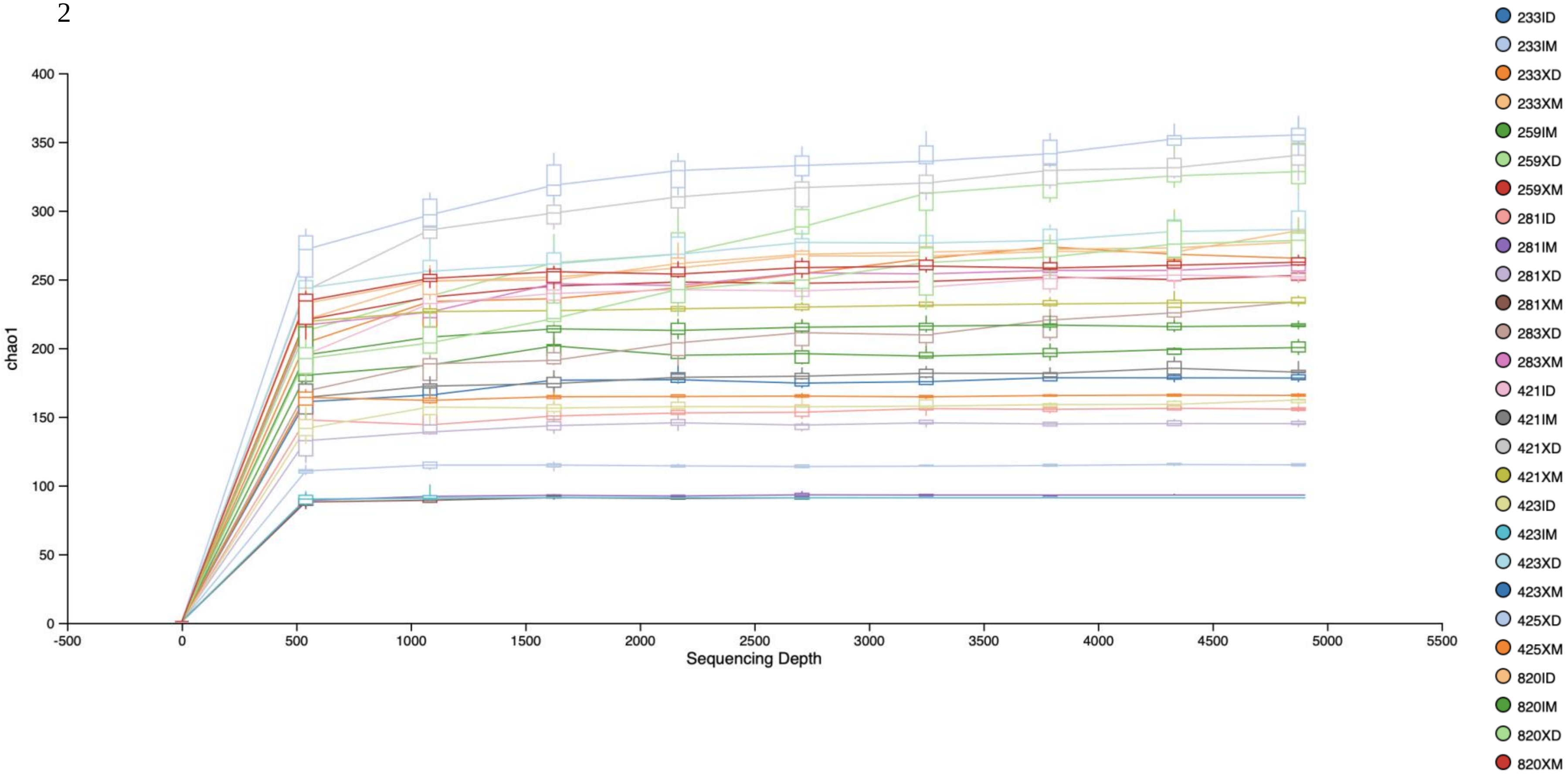

3
4

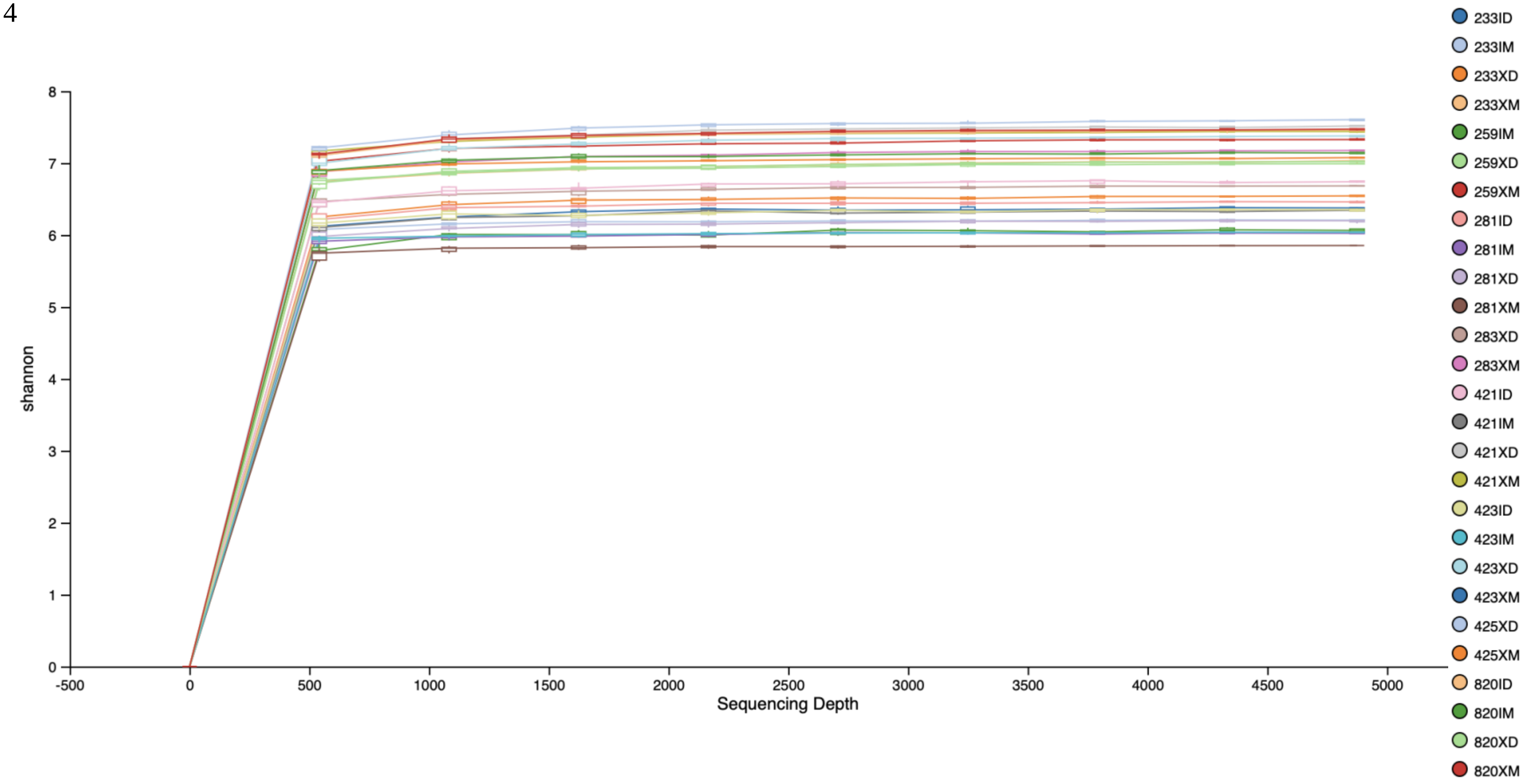
shannon
Sequencing Depth
8
7
6
5
4
3
2
1
0
-500
0
500
1000
1500
2000
2500
3000
3500
4000
4500
5000
233ID
233IM
233XD
233XM
259IM
259XD
259XM
281ID
281IM
281XD
281XM
283XD
283XM
421ID
421IM
421XD
421XM
423ID
423IM
423XD
423XM
425XD
425XM
820ID
820IM
820XD
820XM

Supplementary Figure 3

Table Taxonomic Analysis

ileum mucosa						
**Phylum**	**Family**	**Genus**	**Relative abundance (%)**		**SEM**	**P-value**
			**Uninfected**	**Infected**		
Actinobacteria			7.55	6.74	2.63	0.857
	Corynebacteriaceae		0.56	2.62	0.29	0.854
		Corynebacterium 1	0.49	2.62	0.94	0.582
	Micrococcaceae		0.54		0.19	0.359
	Propionibacteriaceae		1.95	3.50	0.97	0.857
		Cutibacterium	1.93	3.48	0.96	0.857
	Atopobiaceae		2.94		1.08	0.558
		Olsenella	2.48		0.97	0.558
Bacteroidetes			9.07	11.20	3.49	1
	Bacteroidaceae		2.13		0.75	0.372
		Bacteroides	2.11		0.75	0.372
	Marinifilaceae		0.35		0.22	1
		Odoribacter	0.34		0.16	0.845
	Muribaculaceae		2.12	2.08	0.97	1
		uncultured bacterium	1.67		0.85	0.721
	Prevotellaceae		2.65	4.12	1.08	0.475
		Prevotella 1	0.12	1.32	0.47	0.359
		Prevotella 9	0.30		0.19	1
		Prevotellaceae NK3B31 group	0.60		0.22	0.558
		Prevotellaceae UCG-003	0.38	0.31	0.16	1
		uncultured		0.19	0.06	0.582
		Unclassified		0.13	0.07	0.854
	Rikenellaceae		0.85	0.89	0.34	1
		Rikenellaceae RC9 gut group	0.44	0.88	0.24	0.582
	Tannerellaceae		0.66		0.23	0.359
		Parabacteroides	0.65		0.22	0.359
Cyanobacteria			2.97	9.30	2.60	0.229
	Unclassified		0.42		0.20	0.845
		Unclassified	0.42		0.20	0.845
	Eimeria praecox		2.47	8.16	2.36	0.23
		Eimeria praecox	2.47	8.16	2.36	0.23
Epsilonbacteraeota			0.37	1.13	0.34	0.359
	Campylobacteraceae		0.37	1.13	0.34	0.36
		Campylobacter	0.36	1.13	0.34	0.359
Firmicutes			65.40	62.00	5.82	1
	Christensenellaceae		1.04		0.53	0.845
		Christensenellaceae R-7 group	1.03		0.52	0.845
	Clostridiaceae 1		15.05	31.38	10.19	0.629
		Candidatus Arthromitus	13.74	28.22	10.55	0.857
		Clostridium sensu stricto 1	1.11	3.02	1.10	0.582
	Clostridiales vadinBB60 group		0.08		0.06	1
	Family XIII		1.87	1.15	0.65	1
		Family XIII AD3011 group		0.55	0.15	0.359
		Mogibacterium	1.00	0.26	0.49	1
		[Eubacterium] brachy group	0.31		0.14	0.558
		[Eubacterium] nodatum group	0.25		0.11	0.845
		Unclassified	0.25		0.08	0.359
	Lachnospiraceae		14.64	8.17	3.28	0.4
		Butyrivibrio	0.55		0.27	0.845
		Lachnospiraceae NK3A20 group		0.59	0.26	0.435
		Lachnospiraceae UCG-002		0.40	0.15	0.123
		Roseburia	0.11		0.10	1
		Syntrophococcus	2.08		0.98	0.582
		Unclassified	8.04	4.25	1.89	0.629
	Peptostreptococcaceae		9.35	3.91	3.48	0.721
		Romboutsia	8.76	3.23	3.23	0.854
		Unclassified	0.53		0.35	1
	Ruminococcaceae		17.77	11.29	3.81	0.629
		Candidatus Soleaferrea	0.37		0.14	0.27
		Negativibacillus	0.54		0.16	0.582
		Oscillibacter	0.24		0.08	0.119

		Ruminococcaceae UCG-002	0.12		0.15	1
		Ruminococcaceae UCG-004		0.45	0.14	0.123
		Ruminococcaceae UCG-005	5.90	2.34	2.10	0.854
		Ruminococcaceae UCG-009	0.14		0.06	0.27
		Ruminococcaceae UCG-010	2.49		0.98	0.845
		Ruminococcaceae UCG-013	0.23	0.35	0.11	0.854
		Ruminococcaceae UCG-014	0.46		0.21	0.845
		Ruminococcus 2	0.12	0.50	0.14	0.359
		[Eubacterium] coprostanoligenes group	5.35	1.92	1.41	0.229
		Unclassified	0.67	2.06	0.54	0.359
	Erysipelotrichaceae		2.83	4.92	1.95	0.857
		Erysipelotrichaceae UCG-004	0.20		0.08	0.27
		Erysipelotrichaceae UCG-009	0.29	0.15	0.10	0.854
		Turicibacter	0.96		1.66	0.854
		uncultured	0.17		0.07	0.27
Proteobacteria			13.40	8.71	4.76	0.629
	Beijerinckiaceae		0.72		0.25	0.119
	Rhodobacteraceae		0.29		0.16	0.845
		Succinivibrio		1.09	0.45	0.435
	Succinivibrionaceae		0.13	1.09	0.44	0.854
	Burkholderiaceae		0.80		0.42	0.854
		Sutterella	0.74		0.41	1
	Neisseriaceae		1.14		0.50	0.27
	Enterobacteriaceae		9.91	6.36	4.74	0.857
		Escherichia-Shigella	9.84	6.32	4.70	0.629
	Moraxellaceae		0.23		0.08	0.582
Tenericutes			0.82	0.34	0.33	0.854

ileum digesta						
Phylum	Family	Genus	Relative abundance (%)		SEM	P-value
			Uninfected	Infected		
Actinobacteria			2.47		0.97	1
	Atopobiaceae		2.14		0.84	1
		Olsenella	1.69		0.77	1
Bacteroidetes			4.49		4.73	1
	Bacteroidaceae		1.00		0.68	1
		Bacteroides	1.00		0.68	1
	Muribaculaceae		0.51		1.12	1
	Prevotellaceae		1.91		1.38	1
Cyanobacteria			1.07	0.68	0.33	0.80
Firmicutes			77.29	70.73	7.28	0.37
	Christensenellaceae		0.89		1.06	0.8
	Clostridiaceae 1		10.13	4.50	4.79	1
		Candidatus Arthromitus	0.53	0.80	0.22	0.8
		Clostridium sensu stricto 1	9.60	3.71	4.85	1
	Defluviitaleaceae		0.17		0.07	0.333
		Defluviitaleaceae UCG-011	0.17		0.07	0.333
	Family XIII		1.76	2.77	0.73	0.8
		Mogibacterium	1.04		0.67	1
		[Eubacterium] brachy group		0.26	0.05	0.2
		[Eubacterium] nodatum group	0.48	0.42	0.23	1
	Lachnospiraceae		13.86	13.88	2.32	0.8
		Howardella	0.64		0.34	1
		Lachnospiraceae NK3A20 group	0.23		0.41	1
		Syntrophococcus	2.92		1.15	0.4
		[Ruminococcus] gauvreauii group	1.76		0.84	0.4
		Unclassified	5.82	8.29	2.04	0.8
	Peptostreptococcaceae		19.31	7.94	6.22	0.8
		Romboutsia	17.51	6.67	5.67	0.8
		Unclassified	1.40	1.25	0.47	1
	Ruminococcaceae		22.57	26.96	4.62	0.8
		Fournierella	0.15		0.06	0.333
		Ruminococcaceae UCG-005	6.02		3.54	1
		Ruminococcaceae UCG-013	0.27	0.27	0.08	1
		Ruminococcaceae UCG-014	0.67	0.38	0.24	0.8
		Ruminococcus 2		3.61	1.10	0.139
		[Eubacterium] coprostanoligenes group	9.81	7.65	2.67	0.8
		Unclassified	0.51	2.75	0.71	0.2
	Unclassified			0.52	0.17	0.4
		Unclassified		0.52	0.17	0.4
	Erysipelotrichaceae		8.27	11.05	3.84	1
		Erysipelotrichaceae UCG-004	0.13		0.05	0.333
		Solobacterium	0.36		0.20	1
		Turicibacter	6.08	9.99	3.59	0.8
Patescibacteria			0.53		0.19	0.554
	Saccharimonadaceae		0.48		0.19	0.554
Proteobacteria			13.01	13.36	4.87	0.80
	Burkholderiaceae		1.11		1.00	1
	Enterobacteriaceae		11.77	9.22	4.96	0.8
		Escherichia-Shigella	11.74	9.22	4.94	0.8

Rectum mucosa						
**Phylum**	**Family**	**Genus**	**Relative abundance (%)**		**SEM**	**P-value**
			**Uninfected**	**Infected**		
Actinobacteria			7.89		3.34	0.359
Bacteroidetes			17.75	29.59	3.75	0.229
	Bacteroidaceae		4.31	6.90	1.46	0.629
		Bacteroides	4.32	6.90	1.46	0.629
	Marinifilaceae		0.80	0.48	0.23	0.721
		Odoribacter	0.76	0.29	0.20	0.475
	Muribaculaceae		3.00	5.77	1.23	0.229
		uncultured Porphyromonadaceae bacterium		0.56	0.17	0.629
		uncultured bacterium	2.32	5.04	1.13	0.4
	Prevotellaceae		6.16	9.51	1.49	0.4
		Alloprevotella	0.62	2.48	0.73	0.4
		Prevotella 1	0.16	0.56	0.13	0.229
		Prevotella 2	1.47	1.46	0.43	0.629
		Prevotella 9	0.46	1.00	0.21	0.229
		Prevotellaceae NK3B31 group	1.36	1.13	0.38	1
		Prevotellaceae UCG-003	0.79	1.06	0.31	0.4
		uncultured	0.30	0.95	0.30	0.475
		Unclassified		0.78	0.22	0.372
	Rikenellaceae		1.97	2.03	0.48	1
		Alistipes	0.86	0.94	0.20	0.857
		Rikenellaceae RC9 gut group	1.11	1.09	0.30	0.857
	Tannerellaceae		0.92	2.06	0.35	0.229
		Parabacteroides	0.92	2.06	0.35	0.229
	Flavobacteriaceae			0.15	0.06	0.558
Cyanobacteria			1.51	0.71	0.60	0.854
	uncultured bacterium			0.52	0.20	0.558
		uncultured bacterium		0.52	0.20	0.558
Firmicutes			63.63	63.03	3.24	1
	Streptococcaceae		6.98		2.95	0.242
	Christensenellaceae		2.11	3.67	0.75	0.4
		Christensenellaceae R-7 group	2.11	3.67	0.75	0.4
	Clostridiaceae 1		0.20	0.16	0.06	1
		Clostridium sensu stricto 1	0.20	0.16	0.06	1
	Clostridiales vadinBB60 group			0.38	0.12	0.582
		uncultured bacterium		0.38	0.12	0.582
	Defluviitaleaceae		4.38		1.84	0.242
		Defluviitaleaceae UCG-011	4.39		1.84	0.242
	Family XIII		0.34	0.60	0.22	1
		Family XIII AD3011 group	0.29	0.17	0.08	0.475
	Lachnospiraceae		15.20	18.87	3.14	0.857
		Agathobacter		0.48	0.22	0.119
		Blautia	0.17	0.16	0.07	0.854
		Butyrivibrio	0.51	0.54	0.25	1
		Coprococcus 3	0.29		0.10	0.435
		Lachnoclostridium 10		0.45	0.18	0.558
		Lachnospiraceae AC2044 group		0.53	0.18	0.359
		Lachnospiraceae NK4A136 group		0.31	0.11	0.119
		Lachnospiraceae UCG-010		0.81	0.29	0.854
		Roseburia	0.44		0.13	0.123
		Tyzzerella 4		0.58	0.20	0.359
		Unclassified	12.77	13.80	2.86	0.857
	Peptostreptococcaceae		0.27		0.08	0.123
	Ruminococcaceae		27.79	37.02	6.13	0.629
		Candidatus Soleaferrea		0.41	0.22	0.582
		Fournierella	0.24		0.08	0.696
		Negativibacillus	0.53	1.55	0.30	0.057
		Oscillibacter	0.27	0.48	0.08	0.229
		Ruminiclostridium 6		0.37	0.14	0.559
		Ruminiclostridium 9	0.18	0.32	0.09	0.475
		Ruminococcaceae UCG-002	0.38	0.84	0.29	0.854
		Ruminococcaceae UCG-004		0.10	0.04	0.558
		Ruminococcaceae UCG-005	12.76	19.23	4.44	0.629
		Ruminococcaceae UCG-009	0.41	0.42	0.22	0.854
		Ruminococcaceae UCG-010	5.46	5.00	1.10	0.857
		Ruminococcaceae UCG-013	0.57		0.21	0.721

		Ruminococcaceae UCG-014	1.33	1.02	0.46	0.857
		[Eubacterium] coprostanoligenes group	3.32	4.01	0.88	1
		uncultured		0.43	0.11	0.212
		Unclassified	1.48	2.16	0.39	1
	Unclassified			0.46	0.14	0.629
		Unclassified		0.46	0.14	0.629
	Erysipelotrichaceae		0.59	1.80	0.39	0.114
		Erysipelotrichaceae UCG-004		1.03	0.33	0.057
		Solobacterium	0.10		0.03	0.123
		[Anaerorhabdus] furcosa group		0.24	0.15	1
		uncultured		0.15	0.07	0.845
Proteobacteria			5.61	4.76	1.53	1
	uncultured		0.30	0.41	0.16	0.857
		gut metagenome	0.30	0.25	0.11	0.857
	Succinivibrionaceae		0.20	1.91	0.99	0.721
		Succinivibrio	0.20	1.91	0.99	0.721
	Burkholderiaceae		1.26	2.20	0.45	0.4
		Sutterella	1.26	2.20	0.45	0.4
Spirochaetes			0.20	0.36	0.12	1
	Spirochaetaceae		0.20	0.35	0.12	1
		Treponema 2	0.16	0.35	0.13	0.854
Tenericutes			0.98	1.51	0.29	0.629
	gut metagenome			0.94	0.38	0.854
		gut metagenome		0.94	0.38	0.854

Rectum digesta						
Phylum	Family	Genus	Relative abundance (%)		SEM	P-value
			Uninfected	Infected		
Bacteroidetes			19.3625	24.28	3.450129	0.886
	Bacteroidaceae		3.894159087	4.162689279	1.168233	1
		Bacteroides	3.894159087	4.162689279	1.168233	1
	Marinifilaceae		0.5839289	0.289527819	0.132333	0.245
		Odoribacter	0.506959236	0.175038492	0.129741	0.183
	Muribaculaceae		6.097750964	7.34193672	1.865475	1
		uncultured Porphyromonadaceae bacterium		0.847737035	0.363911	0.869
		uncultured bacterium	5.075845228	6.298822757	1.68914	0.886
		Unclassified	0.39492396		0.14902	1
	Prevotellaceae		4.489654792	8.608008956	1.722329	0.343
		Alloprevotella	0.57574133	2.98765317	1.072122	0.486
		Prevotella 1	0.121013681	0.22594707	0.049194	0.686
		Prevotella 2	0.797714226	1.605788794	0.508177	0.561
		Prevotella 9		0.405067207	0.110095	0.309
		Prevotellaceae NK3B31 group	0.502239996	0.589478549	0.161243	1
		Prevotellaceae UCG-003	1.84704742	1.413356832	0.398998	0.886
		uncultured	0.10854826	0.509077939	0.149316	0.2
		Unclassified	0.29782454	0.871639397	0.210865	0.343
	Rikenellaceae		2.463403822	1.720361427	0.521793	0.686
		Alistipes	1.158601847	0.448787953	0.25538	0.245
		Rikenellaceae RC9 gut group	1.304801976	1.271573474	0.338506	1
	Tannerellaceae		1.426802248	1.163602302	0.33654	1
		Parabacteroides	1.426802248	1.163602302	0.33654	1
	uncultured		0.199577758		0.092372	0.869
		uncultured bacterium	0.199577758		0.092372	0.869
Cyanobacteria			0.26	0.1725	0.040309	0.191
	uncultured bacterium		0.193770917	0.116372695	0.051437	0.46
		uncultured bacterium	0.193770917	0.116372695	0.051437	0.46
Firmicutes			73.185	71.905	2.862713	1
	Christensenellaceae		3.408515617	5.498796682	1.381911	0.686
		Christensenellaceae R-7 group	3.408515617	5.498796682	1.381911	0.686
	Clostridiaceae 1		1.108854581	0.137724677	0.42041	0.3
		Clostridium sensu stricto 1	0.742603158	0.137724677	0.259015	0.3
	Clostridiales vadinBB60 group		0.255282496	0.171686723	0.080597	0.882
		uncultured bacterium		0.171686723	0.059756	0.657
	Family XIII		1.176845262	0.619286081	0.277664	0.343
		Family XIII AD3011 group	0.563702009		0.188198	0.46
		[Eubacterium] brachy group	0.052607606		0.020521	0.869
		[Eubacterium] nodatum group	0.350542769		0.116714	0.309
	Lachnospiraceae		16.71442335	19.49568441	2.148406	0.486
		Agathobacter		0.225429389	0.074943	0.408
		Blautia		0.127756612	0.049983	0.186
		Butyrivibrio	0.220754488	0.479259745	0.162142	0.645
		Coprococcus 3	0.08692902		0.149556	0.869
		Dorea		0.172791913	0.063723	0.356
		Lachnoclostridium 10		0.247648854	0.104289	0.62
		Lachnospiraceae AC2044 group		1.251794331	0.370364	0.124
		Lachnospiraceae NK4A136 group		0.573031143	0.226004	0.62
		Lachnospiraceae UCG-010	0.585205461	1.573944426	0.47894	0.686
		Roseburia	0.510669448		0.14049	0.124
		Tyzzerella 4	0.149274329	1.221485477	0.433991	0.645
		Unclassified	11.66683027	12.9276814	1.488806	0.886
	Ruminococcaceae		38.60713954	44.16735124	5.519006	0.686
		Candidatus Soleaferrea	1.230231649	0.768702996	0.421596	0.561
		Fournierella	0.766322427		0.239188	0.3
		Negativibacillus	1.142086918	2.641065332	0.6959	0.343
		Oscillibacter	0.196799371	0.449403083	0.086096	0.191
		Ruminiclostridium 9	0.121353701		0.031758	0.3
		Ruminococcaceae UCG-002	0.919138776	0.843622108	0.270282	1
		Ruminococcaceae UCG-005	16.96592825	23.58553038	4.74098	0.686
		Ruminococcaceae UCG-008	0.083490732	0.178974148	0.069585	1
		Ruminococcaceae UCG-009	0.472121251	0.425165639	0.176254	1
		Ruminococcaceae UCG-010	4.269824524	3.386350483	0.990932	1
		Ruminococcaceae UCG-013	0.824161586	0.454162068	0.185123	0.486
		Ruminococcaceae UCG-014	1.3364725	0.552344705	0.49924	1

		[Eubacterium] coprostanoligenes group	6.979429084	6.938152552	1.221397	0.886
		uncultured	0.16195072	0.275584322	0.071989	0.657
		Unclassified	2.849390188	2.927313848	0.674835	0.886
	Unclassified		0.481825325	0.523724887	0.142696	0.686
		Unclassified	0.481825325	0.523724887	0.142696	0.686
	Erysipelotrichaceae		1.693215173	0.967331981	0.521912	0.686
		Erysipelotrichaceae UCG-004	0.100692938		0.258805	0.657
		[Anaerorhabdus] furcosa group	0.174207962	0.197024277	0.088066	1
		uncultured	0.181525854		0.041874	0.183
Proteobacteria			4.3925	2.565	0.695656	0.486
	uncultured		1.068360058	0.096739235	0.336474	0.183
		gut metagenome	1.005999691	0.082932801	0.32713	0.183
	Succinivibrionaceae		0.095163342	0.429308257	0.136253	0.645
		Succinivibrio		0.429308257	0.139653	0.408
	Burkholderiaceae		1.765172647	1.706221189	0.514899	1
		Sutterella	1.757369088	1.706221189	0.516654	1
	Enterobacteriaceae			0.332526906	0.715607	0.869
		Escherichia-Shigella		0.332526906	0.715607	0.869
Tenericutes			1.2	0.9125	0.416889	1
	gut metagenome		1.074163549	0.755097109	0.449454	0.878
		gut metagenome	1.074163549	0.755097109	0.449454	0.878
